# Structure-function studies of *Vibrio cholerae* quorum-sensing receptor CqsR signal recognition

**DOI:** 10.1371/journal.ppat.1013447

**Published:** 2025-09-04

**Authors:** Andrew M. Guarnaccia, Anjali D. Steenhaut, Sandra D. Olenic, Jesse Na, Lark J. Perez, Wai-Leung Ng, Matthew B. Neiditch

**Affiliations:** 1 Department of Microbiology, Biochemistry, and Molecular Genetics, New Jersey Medical School, Rutgers Biomedical and Health Sciences, Newark, New Jersey, United States of America; 2 Department of Molecular Biology and Microbiology, Tufts University School of Medicine, Boston, Massachusetts, United States of America; 3 Department of Chemistry & Biochemistry, Rowan University, Glassboro, New Jersey, United States of America; 4 Program in Molecular Microbiology, Tufts University, Graduate School of Biomedical Sciences, Boston, Massachusetts, United States of America; Wadsworth Center, UNITED STATES OF AMERICA

## Abstract

Ethanolamine signaling through the transmembrane quorum-sensing receptor CqsR influences *Vibrio cholerae* niche recognition and host colonization. In this study, we present a comprehensive structure-function analysis of CqsR. Specifically, we have determined X-ray crystal structures of the CqsR periplasmic domain bound to the signaling agonist ethanolamine and its analogs, serinol and L-alaninol, as well as the ligand-free (apo) form of CqsR. The periplasmic ligand-binding domain of CqsR is a Cache domain, the most prevalent extracellular sensory module in prokaryotes. Our findings provide a rare structural comparison of ligand-bound and unbound states of a Cache domain receptor. Coupled with thermodynamic binding assays and genetic analyses, these structures elucidate the molecular basis of CqsR ligand specificity. This study not only advances the understanding of Cache domain function but also informs the identification of ligands for orphan Cache receptors and the rational design of signaling agonists and antagonists. Lastly, we discuss ligand-induced conformational changes in the CqsR Cache domains and explore the potential for the existence of additional regulatory ligands.

## Author summary

Quorum sensing, a form of bacterial cell-cell communication, regulates diverse developmental behaviors. This study investigates CqsR, a transmembrane receptor in *Vibrio cholerae*, the gastrointestinal pathogen responsible for cholera, a severe diarrheal disease in humans. CqsR functions by detecting ethanolamine, which enables the bacterium to recognize specific niches within the host. Using X-ray crystallography, we determined structures of the CqsR periplasmic domain alone and in complex with signaling agonists, confirming the classification of CqsR within the Cache domain family—the most widely used extracellular sensory module in prokaryotes. Through structural comparisons, thermodynamic binding assays, mass spectrometry, and genetic analyses, we provide key insights into CqsR ligand specificity. Beyond advancing our understanding of Cache domain function, this work will aid in identifying ligands for orphan Cache receptors and designing signaling modulators. Additionally, we discuss the potential existence of other CqsR regulatory ligands. Our analysis of ligand binding to the most widespread extracellular regulatory domain in prokaryotes will contribute to future antimicrobial strategies targeting bacterial communication pathways.

## Introduction

Quorum sensing systems are widespread in bacteria and enable them to use secreted signals called autoinducers to communicate within and between species [1–4]. Quorum sensing has been extensively studied in the Gram-negative bacterium *Vibrio cholerae,* where it regulates virulence factor production, biofilm formation, Type VI secretion, and, in turn, its ability to cause the diarrheal disease cholera in its human host [5–10].

**Fig 1:**
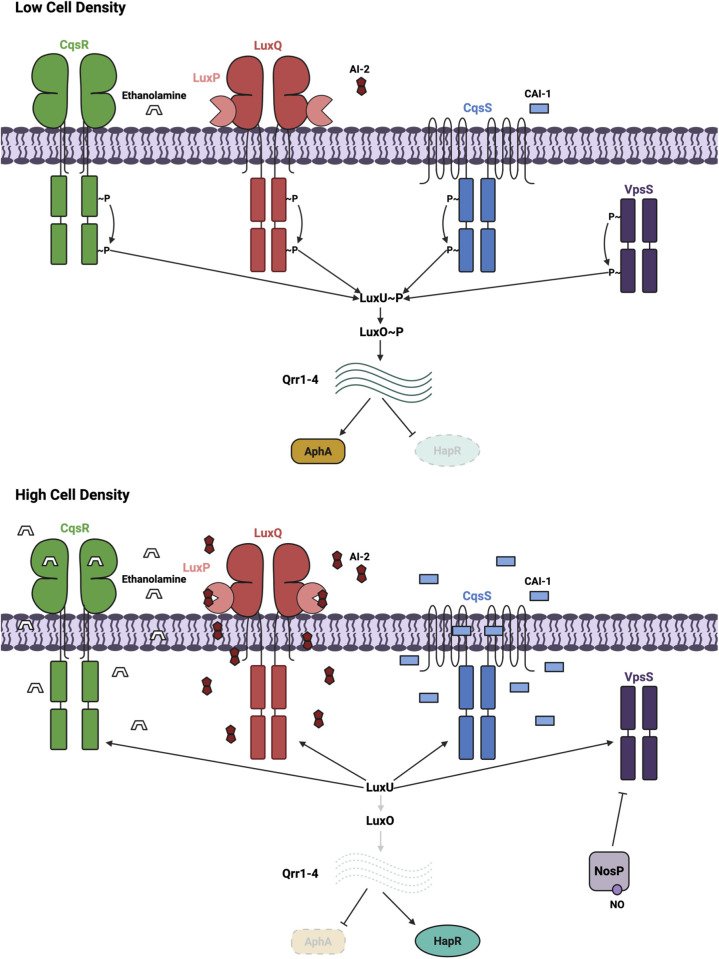
Cartoon schematic of quorum sensing in *Vibrio cholerae.* Quorum sensing in *V. cholerae* is regulated by four receptor histidine kinases: CqsR, LuxPQ, CqsS, and VpsS. Top: Low-cell density signaling. All four QS receptors act as histidine kinases, phosphorylating LuxU, which transfers a phosphoryl group to LuxO and leads to transcription of Qrr1-4. These small RNAs inhibit HapR and induce AphA production, respectively. Bottom: High-cell density signaling. The QS receptors bind their respective autoinducers, inhibiting receptor kinase activity. This causes phosphoryl group transfer away from LuxO, resulting in decreased Qrr1-4 transcription and promoting HapR production. Created in BioRender. Guarnaccia, A. (2025) https://BioRender.com/tfg4c8z.

The canonical *V. cholerae* quorum sensing circuit is controlled by four histidine kinases CqsR, CqsS, LuxPQ, and VpsS [6,11,12] (Fig 1). Below a threshold level of autoinducers, the kinases phosphorylate the intermediate response regulator protein LuxU [13]. LuxU transfers phosphoryl groups to LuxO, driving the transcription of the small regulatory RNAs, Qrr1–4 [11,14]. These RNAs activate and repress the translation of AphA and HapR, respectively, resulting in a low cell density gene expression pattern [15,16]. Above the threshold level of autoinducers, the kinase activity of the quorum-sensing receptors is inhibited, and Qrr1–4 transcription is reduced. Consequently, HapR translation is induced, AphA translation is repressed, and *V. cholerae* adopts a high cell density gene expression pattern [14,17]. Importantly, mutants lacking LuxO, the four QS receptors, or the four Qrr sRNAs are avirulent [6,11,12]. In addition, there is another QS system in *V. cholerae* that does not control LuxO [18], and its role in host colonization is unknown.

CqsS and LuxPQ are regulated by the bacterial quorum sensing signals *S*-3-hydroxytridecan-4-one (CAI-1) and *S*-TMHF-borate (AI-2), respectively [19–28]. VpsS phosphorylates LuxU *in vitro*, and this activity is inhibited by a regulatory protein NosP in complex with nitric oxide [29]. *V. cholerae* does not make nitric oxide, and a NosP deletion had no effect on quorum sensing; therefore, the role of NO in *V. cholerae* sensing remains unknown. CqsR, however, is regulated by ethanolamine, which is produced by *V. cholerae* and the host [30,31]. Each of the four *V. cholerae* quorum-sensing receptors is alone sufficient to activate Qrr transcription and support host colonization [30]. The functional redundancy of these QS receptors is essential for preventing interference by environmental signals, avoiding premature commitment to the high-cell density quorum-sensing program that could shut off virulence and biofilm gene expression.

It is important to note that, unlike CAI-1 and AI-2, which are bona fide autoinducers, the quantity of ethanolamine produced by *V. cholerae* alone is not sufficient to regulate its quorum-sensing response [30]. Furthermore, our genetic results suggest that molecule(s) produced by *V. cholerae* may act on CqsR. However, the quantity of ethanolamine in certain environments, such as the distal part of the GI tract, is sufficient to regulate CqsR signaling, and we showed that *V. cholerae* colonization here is disrupted in a mutant strain where CqsR is the only quorum-sensing receptor [11,30,32].

Despite their critical importance to *V. cholerae* quorum sensing and pathogenesis, so far, only structural studies of LuxP and the LuxPQ periplasmic complex have been conducted [26–28]. Chemical genetic approaches have been used to identify residues important for signal recognition of CAI-1 in CqsS, whose multipass transmembrane region makes it a challenging target for structural analysis [20,21]. VpsS is an orphan receptor, i.e., its regulatory ligand is unknown. In contrast, we have shown that CqsR is regulated by ethanolamine as well as the less common metabolites serinol and L-alaninol [30]. Here, we present structure-function analysis of CqsR, revealing the mechanistic basis of its ligand-binding specificity. We have determined the X-ray crystal structure of the CqsR periplasmic domain (CqsR_p_) alone and in complex with ethanolamine. Furthermore, we carried out thermodynamic binding studies of CqsR_p_ with ethanolamine and its analogs, and we solved the X-ray crystal structure of CqsR_p_ in complex with the tightest-binding ethanolamine analogs L-alaninol and serinol. We also studied the role of ligand-binding site residues using biochemical and genetic studies.

As anticipated based on sequence analysis, our structural studies confirmed that CqsR_p_ is a double Cache domain, which is the most common extracellular ligand-binding domain in bacterial receptors, including, among others, histidine kinases, methyl-accepting chemotaxis proteins, phosphatases, cyclases, and phosphodiesterases [33]. The ligand-bound CqsR_p_ structures show how Cache domains bind ethanolamine and other 2-amino alcohols. The CqsR structures presented here will serve as the archetype for studies of Cache domains that bind similar 2-amino alcohols. With this in mind, we contextualize our findings within the framework of previous elegant studies of Cache domain evolution, ligand-binding specificity, and molecular recognition. Together, these data from CqsR and other Cache domain ligand-binding studies can aid in the identification of ligands for orphan receptors and guide the rational design of signaling agonists and antagonists targeting receptor Cache domains. Finally, we discuss the ligand-induced conformational changes in the CqsR Cache domains and provide rationale for the existence of additional CqsR regulatory ligands.

## Results

### X-ray crystal structure of CqsR_p_ complexed with ethanolamine

**Fig 2:**
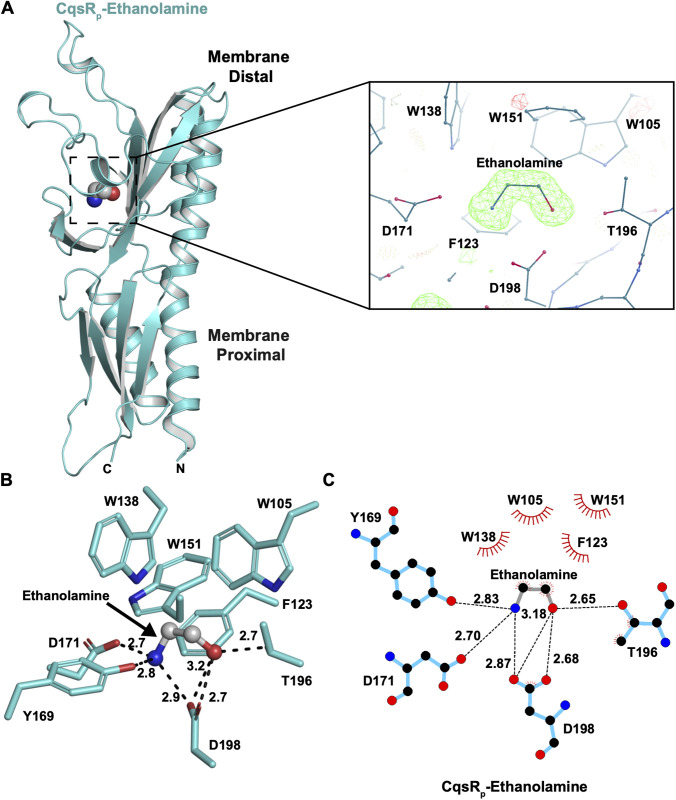
CqsR_p_-ethanolamine crystal structure. (A) Left: The CqsR_p_ monomer and ethanolamine are rendered as a cyan cartoon and spheres, respectively. The schematic was produced using PyMOL (PyMOL Molecular Graphics System, Version 3.0 Schrödinger, LLC). Right: Expanded view of the area enclosed by the box in the left panel. F_o_-F_c_ electron density corresponding to ethanolamine scaled to 3σ. Positive electron density is shown in green and negative density shown in red. F_o_-F_c_ electron density was calculated prior to ligand and water building. The schematic was produced using Coot [34]. (B) Ethanolamine (ball-and-stick model) and its coordinating residues (cyan sticks) are shown. The schematic was produced using PyMOL (PyMOL Molecular Graphics System, Version 3.0 Schrödinger, LLC). (C) Schematic representation of CqsR_p_-ethanolamine binding. CqsR_p_ and ethanolamine are depicted as blue and grey bonds, respectively. Hydrogen bonds are depicted as black dashed lines. Hydrophobic contacts are depicted as lines radiating from the semicircles and spheres. The schematic was produced using LigPlot+ [35].

To understand the mechanistic basis of ethanolamine recognition by CqsR, we determined the 1.55 Å resolution X-ray crystal structure of the CqsR periplasmic domain (CqsR_p_) in complex with ethanolamine (Fig 2 and Table 1). To obtain this structure, CqsR_p_ crystals were soaked in cryoprotectant containing ethanolamine (S1 Table). However, it is important to note that subsequent LC-MS/MS analysis revealed that 103.3 μM CqsR_p_ is bound to 83.9 μM ethanolamine following overexpression in *E. coli* and purification (S1 Fig and S2 Table). Ultimately, we also obtained identical crystal structures without supplementing the cryoprotectant with ethanolamine and without adding it during crystallization.

**Table 1:** Data collection and refinement statistics.

	CqsR_p_-ethanolamine	CqsR_p_-L-alaninol	CqsR_p_-serinol	CqsR_p_-D198N
**PDB ID**	9NIA	9NIT	9NIV	9NJ8
**Data collection**				
Space group	I 41 2 2	I 41 2 2	I 41 2 2	P 31 2 1
Cell dimensions				
a, b, c (Å)	121.81, 121.81, 70.71	121.02, 121.02, 70.55	121.14, 121.14, 70.82	111.71, 111.71, 75.74
α, β, γ (°)	90.0, 90.0, 90.0	90.0, 90.0, 90.0	90.0, 90.0, 90.0	90.0, 90.0, 120.0
Resolution (Å)	50.00-1.55 (1.58-1.55)	19.69-1.72 (1.78-1.72)	21.64-1.75 (1.81-1.75)	19.88-2.50 (2.59-2.50)
Wavelength (Å)	0.97946	1.540562	1.540562	1.540562
Completeness (%)	100 (100)	95.5 (76.1)	99.7 (97.9)	99.7 (99.9)
CC_1/2_	0.997 (0.560)	0.999 (0.670)	0.999 (0.585)	0.999 (0.760)
Average I/ σI	5.80 (1.01)	7.26 (1.21)	7.32 (1.57)	6.38 (1.37)
Redundancy	12.6 (8.3)	8.7 (3.4)	8.8 (3.6)	12.7 (13.4)
Total reflections	488,909	233,960	236,409	243,812
Unique reflections	38,762	26,788	26,724	19,154
**Refinement**				
R_work_/ R_free_	0.1831 (0.2971)/ 0.2075 (0.3287)	0.1796 (0.3037)/ 0.2204 (0.3334)	0.1847 (0.2977)/ 0.2212 (0.3178)	0.2171 (0.3610)/ 0.2628 (0.3878)
**Number of atoms**				
All atoms	1864	1904	1894	3131
Protein	1722	1702	1720	3122
Ligands	5	6	7	0
Water	137	196	173	8
**Average B-factor (Å**^**2**^)				
All atoms	34.60	25.97	26.42	69.18
Protein	34.36	25.31	26.10	69.23
Ligands	21.97	13.53	17.66	0
Water	38.15	32.06	29.63	48.97
**R.m.s. deviations**				
Bond lengths (Å)	0.010	0.006	0.007	0.004
Bond angles (°)	0.933	0.807	0.924	0.556
**Ramachandran statistics**				
Favored (%)	99.05	99.05	98.08	94.64
Allowed (%)	0.95	0.95	1.92	5.36
Outliers (%)	0	0	0	0

Data collection and refinement statistics. R_sym_ = Σ_h_ Σ_i_ | I_i_(h) - < I(h)> |/ Σ_h_ Σ_i_ I_i_(h), where I_i_(h) is the i^th^ measurement of h and <I(h)> is the mean of all measurements of I(h) for reflection h. R_work_ = Σ ||F_o_| - |F_c_||/ Σ |F_o_|, calculated with a working set of reflections. R_free_ is R_work_ calculated with only the test set of reflections (CqsR_p_-ethanolamine, 5.16%; CqsR_p_-L-alaninol, 10.00%; CqsR_p_-serinol, 7.61%; CqsR_p_-D198N, 10.31%). Data for the highest resolution shell are given in parentheses. The structures were determined using single crystals.

Consistent with sequence analysis [30], structural comparison of CqsR_p_ to all structures in the PDB using Dali showed that it forms a double Cache domain (Fig 2A) [36]. Electron density corresponding to ethanolamine revealed that it binds to the core of the membrane distal domain (Fig 2A). The ethanolamine binding site consists of a hydrophilic platform and a hydrophobic lid (Fig 2B and 2C). More specifically, the acidic side chain of CqsR-D198 plays a critical role, serving to coordinate both the ethanolamine amine and hydroxyl. As both ethanolamine functional groups are chelated by Asp198, this enforces a higher energy gauche configuration for the ligand. CqsR residues Tyr169, Asp171, and Thr196 mediate additional contacts with ethanolamine. The aromatic hydrophobic side chains of residues Trp105, Phe123, Trp138, and Trp151 form a lid above ethanolamine, with the face of Trp151 prominently positioned to engage in hydrophobic contacts with the ethanolamine alkane carbon atoms.

### X-ray crystal structure of apo CqsR_p_ (CqsR_p_-D198N)

Because purified CqsR_p_ contains ethanolamine derived from the *E. coli* overexpression host, to obtain an apo, i.e., ligand-free, structure, we generated CqsR_p_-D198N. Based on the CqsR_p_-ethanolamine structure described above, we hypothesized that substituting Asp198 for Asn would disrupt Asp198 hydrogen bonding with ethanolamine without disrupting protein folding (Fig 2B and 2C). LC-MS/MS analysis of CqsR_p_-D198N showed that it contains less than 2.1 ± 0.2 μM ethanolamine in a 103.3 μM sample of protein, and we then determined the X-ray crystal structure of apo CqsR_p_-D198N to 2.50 Å resolution (Table 1, S1 and S2 Figs, and S3 Table). Since substituting negatively charged Asp198 for uncharged Asn resulted in a dramatic reduction in CqsR_p_-ethanolamine binding affinity as determined by LC-MS/MS and the absence of ethanolamine in the crystal structure, we conclude that the Asp198 negative charge is critical for ethanolamine coordination.

### Thermodynamic binding studies of CqsR to ethanolamine and ethanolamine analogs

**Fig 3:**
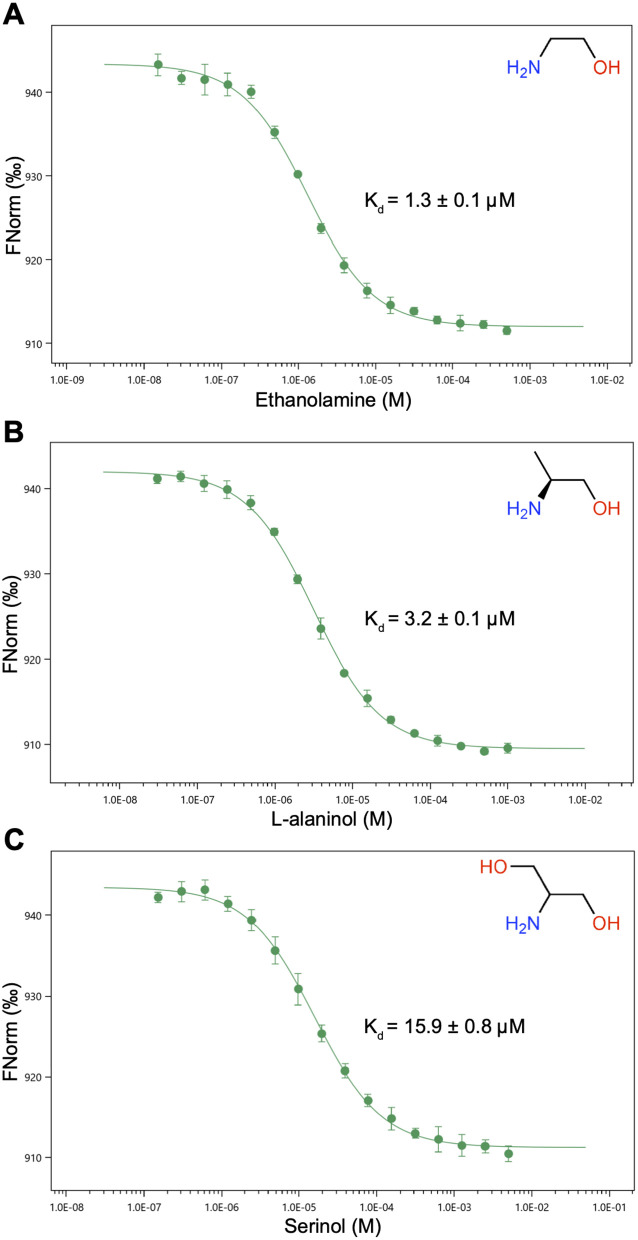
MST quantification of CqsR_p_ binding to ethanolamine, L-alaninol, and serinol. (A) Ethanolamine was titrated between 500.0 μM and 15.3 nM with 100.0 nM CqsR_p_. MST was performed in triplicate. (B) L-alaninol was titrated between 1.0 mM and 30.5 nM with 100.0 nM CqsR_p_. MST was performed in quadruplicate. (C) Serinol was titrated between 5.0 mM and 152.6 nM with 100.0 nM CqsR_p_. MST was performed in quadruplicate.

**Fig 4:**
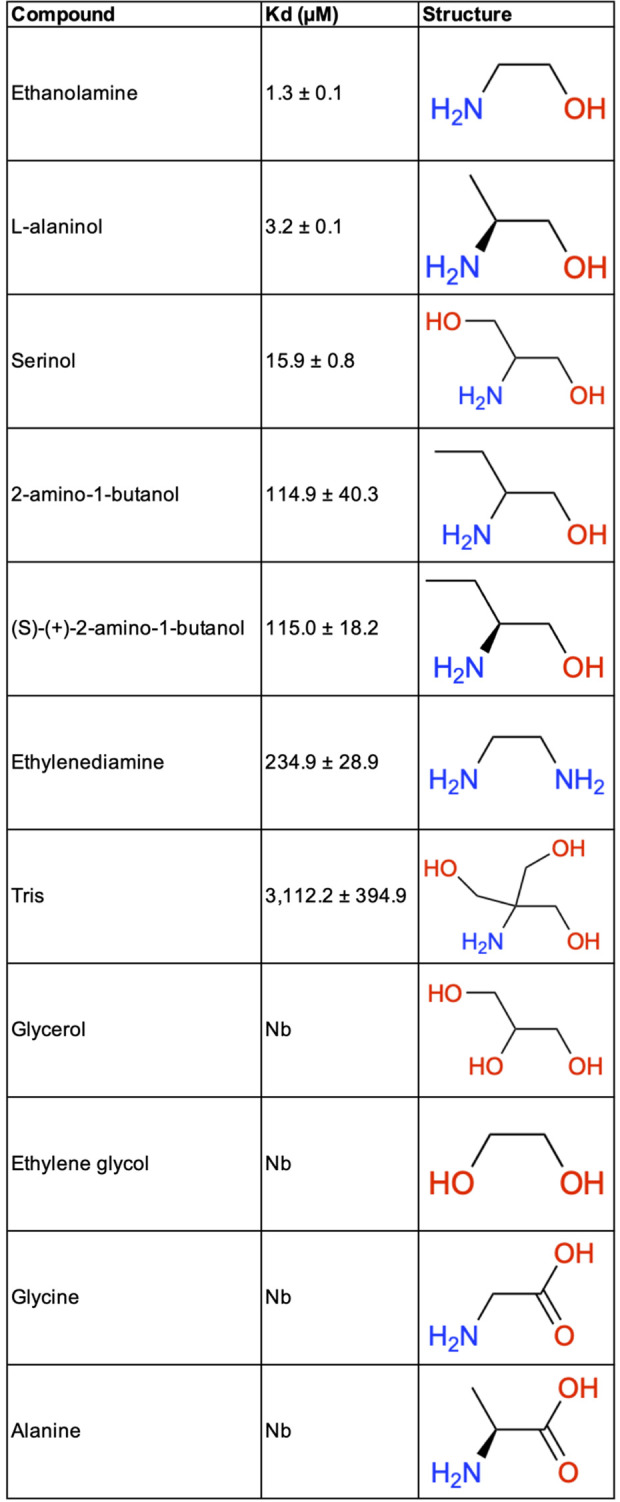
Dissociation constants derived from MST studies of CqsR_p_ binding to ethanolamine analogs. Reported dissociation constants (K_d_) for ethanolamine and compounds of similar chemical composition. Diagrams of each respective structure are shown. Nb indicates that no binding was detected.

We previously tested the common metabolites in the Biolog PM1–4 screens for their ability to bind CqsR_p_ as a function of its thermal unfolding [30]. Ethanolamine, L-alaninol, and serinol increased the CqsR_p_ melting temperature. These structurally similar 2-amino alcohols also acted as CqsR signaling agonists *in vivo*. To quantify their binding to CqsR, we used microscale thermophoresis (MST) (Fig 3). The dissociation constants (K_d_) for CqsR_p_ binding to ethanolamine, L-alaninol, and serinol are 1.3 ± 0.1 μM (Fig 3A), 3.2 ± 0.1 μM (Fig 3B), and 15.9 ± 0.8 μM (Fig 3C), respectively. Importantly, ethanolamine, L-alaninol, and serinol did not bind CqsR_p_-D198N (S3 Fig). In addition, we used MST to evaluate CqsR_p_ binding to additional diverse ethanolamine analogs (Fig 4). Most of these compounds exhibited no detectable binding to CqsR_p_ or otherwise bound very weakly (e.g., ethylenediamine K_d _= 234.9 ± 28.9 μM, 2-amino-1-butanol K_d_ = 114.9 ± 40.3 μM, (S)-(+)-2-amino-1-butanol K_d_ = 115.0 ± 18.2 μM, and tris K_d _= 3,112.2 ± 394.9 μM) compared to ethanolamine, L-alaninol, and serinol (Figs 4 and S4).

### X-ray crystal structures of CqsR in complex with the ethanolamine analogs L-alaninol or serinol

**Fig 5:**
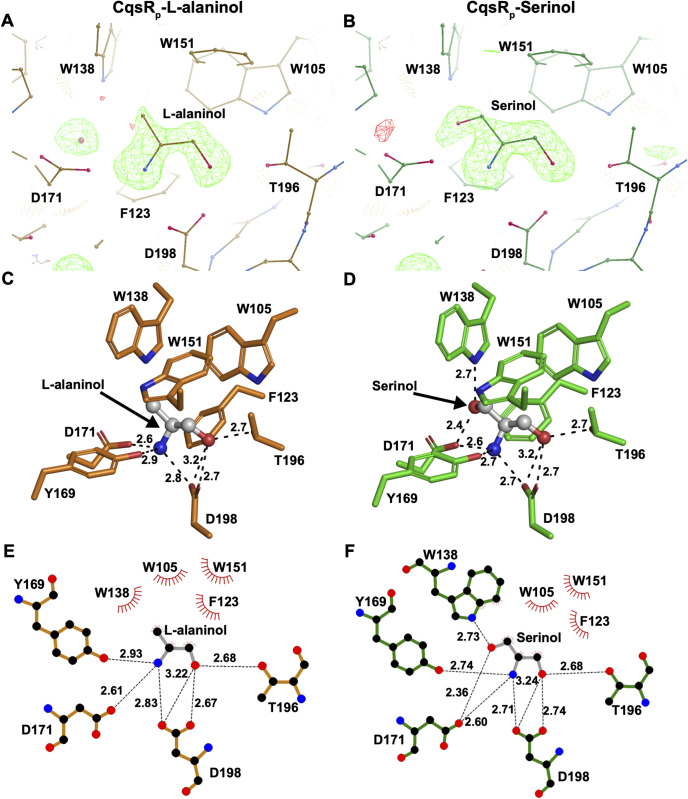
CqsR_p_-L-alaninol and CqsR_p_-serinol binding sites. (A and B) CqsR_p_-L-alaninol and CqsR_p_-serinol binding-site residues are depicted as balls and sticks. F_o_-F_c_ maps scaled to 3σ with positive density shown in green and negative density shown in red. F_o_-F_c_ electron density was calculated prior to ligand and water building. The schematic was produced using Coot [34]. (C and D) Isolated views of the respective ligand binding sites. CqsR residues that coordinate L-alaninol or serinol are shown as orange or green sticks, respectively. Ligands are shown as ball-and-stick models. Note the acidic platform residue Asp198, the hydrophilic residues below the ligands, and the hydrophobic residues forming the lid above. The schematic was produced using PyMOL (PyMOL Molecular Graphics System, Version 3.0 Schrödinger, LLC). (E and F) Schematic representation of CqsR_p_-L-alaninol and CqsR_p_-serinol binding. CqsR_p_ and L-alaninol are depicted as orange and gray bonds, respectively, in panel E. CqsR_p_ and serinol are depicted as green and gray bonds, respectively, in panel F. Hydrogen bonds are depicted as black dashed lines. Hydrophobic contacts are depicted as lines radiating from the semicircles and spheres. The schematic was produced using LigPlot+ [35].

To understand how CqsR binds ethanolamine analogs, we determined the X-ray crystal structures of CqsR_p_-L-alaninol and CqsR_p_-serinol to 1.72 Å and 1.75 Å resolution, respectively (Table 1). In both cases, there is interpretable electron density corresponding to the entire ligand (Fig 5A and 5B). The L-alaninol and serinol atoms analogous to ethanolamine bound to CqsR_p_ in an identical fashion (Figs 2B, 2C and 5C–5F). More specifically, the amine and hydroxyl common to ethanolamine, L-alaninol, and serinol similarly hydrogen bond to the platform residue Asp198. Additional hydrogen bonds to the common amine and hydroxyl are mediated by Tyr169, Asp171, and Thr196 (Fig 5E and 5F). As in the CqsR_p_-ethanolamine structure, aromatic hydrophobic side chains form a lid above the L-alaninol and serinol alkane carbon atoms. Relative to its position in the CqsR_p_-ethanolamine structure, CqsR_p_ Trp138 shifts 0.7 Å and 0.6 Å to accommodate the L-alaninol methyl and serinol methoxy, respectively (Figs 2B, 5C, 5D and S5). Serinol binding also forms hydrogen bonds with Trp138 and Asp171 that are otherwise absent in the CqsR_p_ complexes with ethanolamine and L-alaninol (Fig 5D and 5F). The versatility of Trp138 is notable because it mediates hydrophobic contacts with ethanolamine (Fig 2B and 2C) and L-alaninol (Fig 5C and 5E) but forms a hydrogen bond with serinol (Fig 5D and 5F).

The X-ray crystal structures of CqsR_p_-ethanolamine, CqsR_p_-L-alaninol, and CqsR_p_-serinol in combination with the thermodynamic binding studies of CqsR_p_ to ethanolamine and ethanolamine analogs points to specific requirements for ligand binding to CqsR. Namely, the presence of both an amine positioned proximal for coordination by Asp171/Tyr169, and a hydroxyl oriented towards Thr196 are required for tight binding. Both of these key heteroatom functional groups of the ligand are chelated by Asp198, supporting the observed preference for a ligand with a two-carbon linker between these atoms. Furthermore, the structures and binding studies show where structural modifications in the ligand are tolerated. This is mainly in the observed binding of small α-amino substituents, including methyl and hydroxymethyl, as represented by the ligands L-alaninol and serinol, respectively. Weak CqsR binding is observed for ethylenediamine, in which the hydroxyl of ethanolamine bound by Thr196 is replaced by an amine, tris in which an additional α-amino substituent is incorporated, and both 2-amino-1-butanol and (S)-(+)-2-amino-1-butanol, in which an ethyl functional group replaces the L-alaninol methyl substituent (Fig 4). No binding is observed for ligands in which the amine functionality of the ligand is modified as in glycerol, ethylene glycol, and propanol (Fig 4). Furthermore, replacement of the hydroxyl group with a carboxylic acid (i.e., glycine and alanine), thiol (cysteamine), amide (glycinamide) or removal of this functionality (i.e., propylamine and pentan-3-amine) is not tolerated (Fig 4).

### Structural conservation of the ethanolamine binding site

In addition to CqsR, three other periplasmic ethanolamine-binding receptors have been identified. Two of these proteins, *Desulfovibrio inopinatus* WP_027185430.1 and *Peptoclostridium littorale* WP_052635864.1, previously referred to as R3 and R7, respectively, were shown to bind ethanolamine in an isothermal titration assay [37]. In line with the binding affinity of CqsR_p_ and ethanolamine, R3 bound ethanolamine with K_d _= 2.3 ± 0.1 μM and R7 bound ethanolamine with K_d _= 1.7 ± 0.02 μM. The R3 and R7 periplasmic domains (R3_p_ and R7_p_) are Cache domains whose structures are predicted to be similar to CqsR_p_ as determined by sequence analysis and structure prediction using AlphaFold 3 (S6 Fig) [38]. Based on sequence conservation in the ethanolamine binding site, we predict that ethanolamine binding to R3 and R7 will be structurally similar to that of CqsR (S6 Fig). Namely the use of a platform acidic residue (R3-Asp186 and R7-Asp207) to bind the ethanolamine amine and hydroxyl, and the use of aromatic side chains to form a hydrophobic lid above the ethanolamine alkane atoms (S6 Fig).

In addition, the solute-binding protein Csal_0678 from *Chromohalobacter salexigens* binds ethanolamine, and the Csal_0678 X-ray crystal structure was determined in complex with ethanolamine to 1.40 Å resolution (PDB ID 4UAB) [39]. While there is no recognizable similarity between the tertiary structures of Csal_0678 and CqsR, R3, and R7, i.e., Csal_0678 is not a Cache domain, their ethanolamine binding sites are remarkably similar (S6 Fig). More specifically, Csal_0678 uses a platform acidic residue (Glu220) to coordinate the ethanolamine amine and hydroxyl in an analogous manner to the CqsR, R3, and R7 platform Asp and surrounds the ethanolamine alkane atoms with aromatic side chains.

### Targeted mutagenesis of the CqsR ligand-binding site

Guided by the ligand-bound CqsR_p_ structures, we predicted that Trp151, Tyr169, Asp171, Thr196, and Asp198 are important for ethanolamine binding to CqsR. To test the role of these residues in CqsR signal recognition inside the bacterial cell, we introduced Trp151Ala (W151A), Tyr169Ala (Y169A), Asp171Ala (D171A), Thr196Ala (T196A), or Asp198Asn (D198N) mutations into CqsR of a strain where the other three QS receptors are absent. This strain also carries a P_*qrr*4_-*luxCDABE* transcriptional reporter so we can measure bioluminescence at different cell densities to monitor quorum-sensing (*qrr*4 transcription) in response to different concentrations of exogenously added ethanolamine.

**Fig 6:**
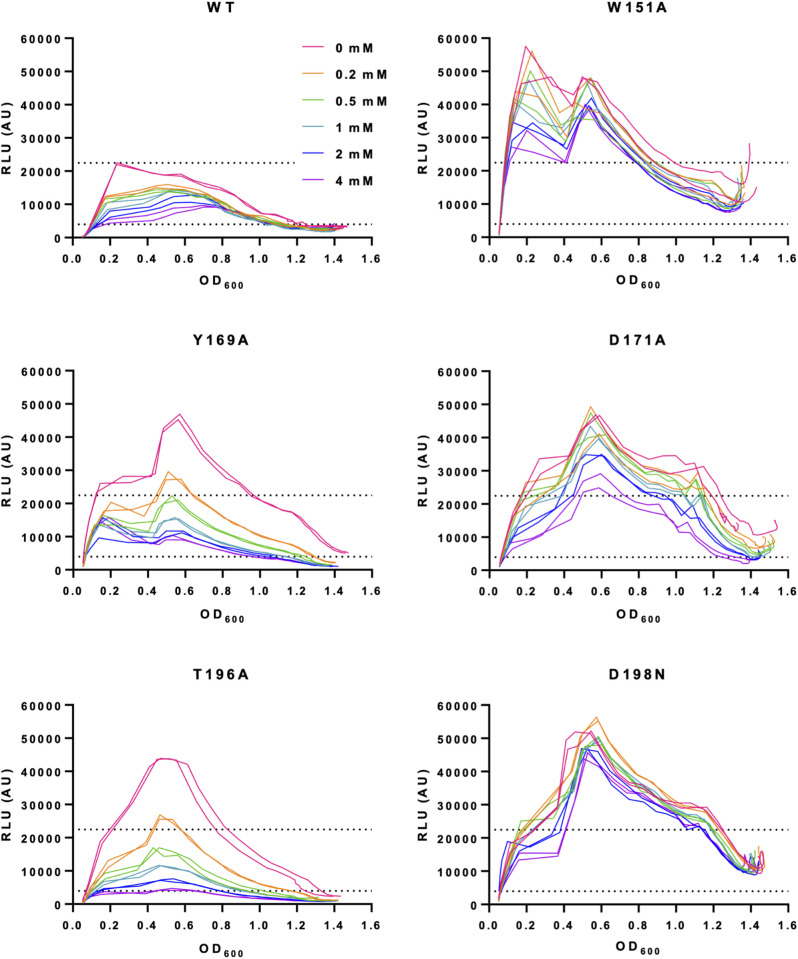
Response to exogenously added ethanolamine by different CqsR variants in *V. cholerae.* Whole-cell bioluminescence assays were performed using a ∆*cqsS* ∆*luxPQ* ∆*cqsR* ∆*vpsS* strain with different CqsR variants expressed from a plasmid. These strains also contain a P_*qrr*4_-*luxCDABE* reporter to measure the transcription of *qrr*4. Ethanolamine was added at the final concentrations as shown. Curves of the same color represent bacterial cultures grown with the same concentration of ethanolamine. For comparison, the two dotted lines on each graph show the maximum light production level of WT in the absence of ethanolamine and the minimal light production level of WT in the presence of the highest amount of ethanolamine at low cell density (OD_600_ ~ 0.2). Representative results are shown with technical duplicates. The experiments have been repeated at least three times. In S7 Fig, these data for mutant CqsR are presented in comparison to the strain containing WT CqsR using a Y-axis scale that improves the visibility of the dose-response curves. In S8 Fig, biological replicates with technical triplicates are shown.

Without any exogenously added ethanolamine, the strain with WT CqsR showed a high level of *qrr*4 transcription at low cell density (OD_600_ ~ 0.2-0.4) and a low level of *qrr*4 transcription at high cell density (OD_600_ > 0.8), as indicated by the increase and the subsequent decrease of bioluminescence during growth. The decrease of *qrr*4 transcription is due to the inhibition of CqsR activity by an unknown native autoinducer, i.e., an autoinducer produced by *V. cholerae*. In contrast, the level of *qrr*4 transcription was reduced in a dose-dependent manner by exogenously added ethanolamine (Figs 6, S7 and S8).

The *qrr*4 transcription pattern in the CqsR-Y169A or -T196A mutant was slightly different from that in the WT. In the absence of exogenously added ethanolamine, these two strains in general produced higher bioluminescence, suggesting the intrinsic CqsR kinase activity in these mutants is higher, possibly due to the inability of the natively made autoinducer to bind to the mutant receptors. However, in both mutants, *qrr*4 transcription was reduced to a level comparable to that of the WT by exogenously added ethanolamine (Figs 6, S7 and S8). Similarly, CqsR-W151A, -D171A, and -D198N produced higher bioluminescence than WT in the absence of exogenously added ethanolamine. However, CqsR-W151A and -D171A displayed an intermediate phenotype, responding to the highest concentrations of ethanolamine but never reducing bioluminescence comparable to that of the WT CqsR strain. In contrast, CqsR-D198N was completely irresponsive to ethanolamine.

These results are consistent with those of our previous genetic screen, where CqsR-D171V and CqsR-D198V resulted in a dramatic loss of signaling sensitivity to the native CqsR autoinducer [30]. The results of our site-directed and random mutagenesis confirm the *in vivo* relevance of the ligand-bound CqsR_p_ X-ray crystal structures and suggest that the ethanolamine-binding residues are also important for the recognition of the native autoinducer.

### Insights into CqsR regulation by ethanolamine, serinol, and L-alaninol

**Fig 7:**
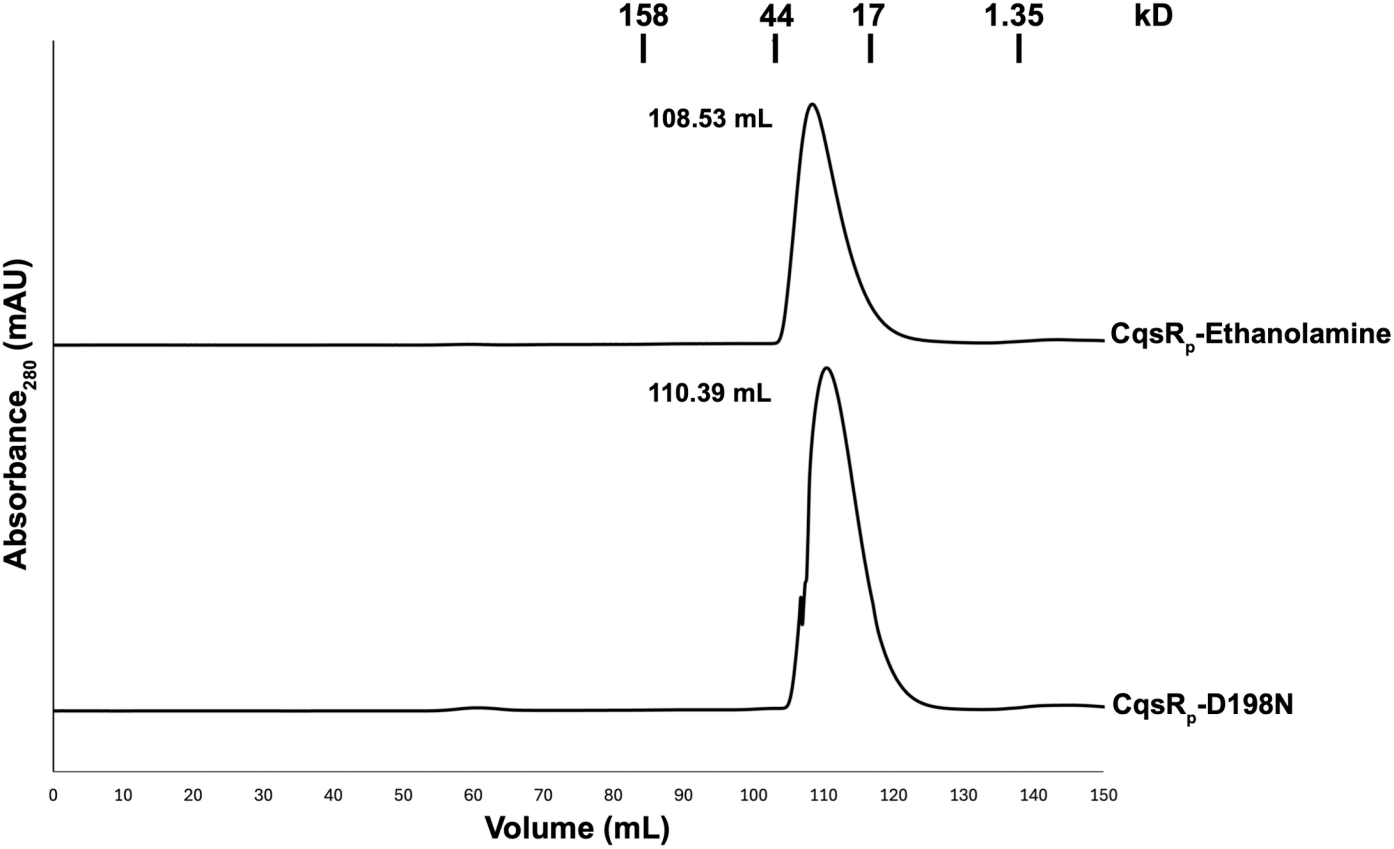
CqsR_p_-ethanolamine and CqsR_p_-D198N are monomeric in solution. Size exclusion chromatography of CqsR_p_-ethanolamine and CqsR_p_-D198N, with peak volumes indicated. CqsR_p_-ethanolamine (MW_theor_ = 24.3 kD, MW_exp_ = 22.8 kD). CqsR_p_-D198N (MW_theor_ = 24.2 kD, MW_exp_ = 19.3 kD). Vertical lines above the absorbance traces indicate protein size standards elution volumes (peak positions). Absorbance was measured at 280 nm and reported as milliabsorbance units (mAU).

Histidine kinases are dimers whose interaction is mediated by their cytoplasmic dimerization and histidine kinase domains (DHp domains) [40–43]. Analytical size-exclusion chromatography of CqsR_p_-ethanolamine and apo CqsR_p_-D198N showed that they are monomeric in solution (Fig 7). Consistent with these results, there is only one protomer in the CqsR_p_-ethanolamine, CqsR_p_-serinol, and CqsR_p_-L-alaninol crystallographic asymmetric units. The application of crystallographic symmetry revealed that none of the symmetry-mates formed physiologically relevant dimers, i.e., interactions positioning their N- and C-termini on the same face in a manner consistent with CqsR_p_ dimer formation in the *V. cholerae* inner membrane. In addition, while the CqsR_p_-D198N crystallographic asymmetric unit was dimeric, the protomers were anti-parallel, which is inconsistent with a biological arrangement. Together, the solution studies and crystallographic analysis suggest that signaling through CqsR is not regulated by ligand-induced shifts in its monomer-dimer equilibrium. Thus, we propose CqsR activity is regulated through the individual monomers within a presumed signaling dimer. It is important to note that this *in vitro* analysis was carried out in the absence of the CqsR transmembrane domain and membrane. It is possible that in the presence of these elements (or the *V. cholerae* autoinducer), CqsR_p_ forms interdimer contacts.

**Fig 8:**
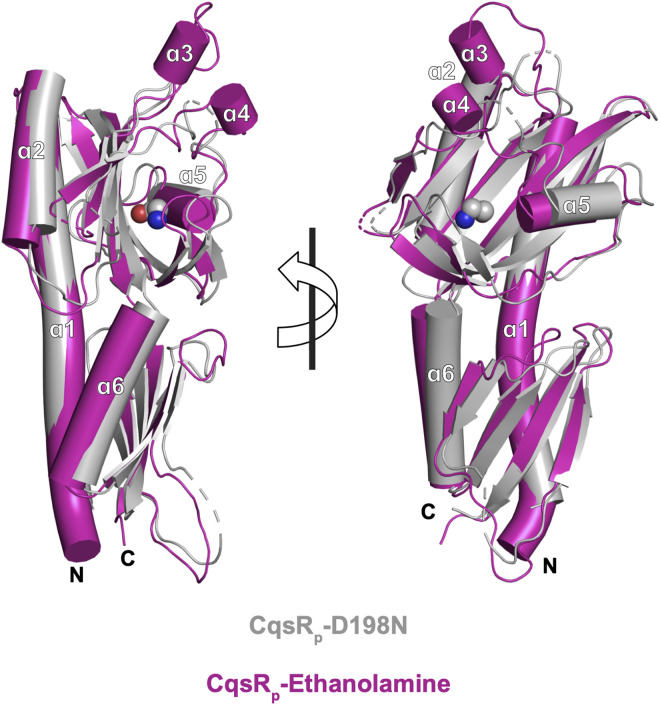
Ethanolamine-induced CqsR_p_ conformational changes. Two different views of apo CqsR_p_-D198N (gray cartoon) and CqsR_p_-ethanolamine (magenta cartoon) aligned using their α1 helices (residues 44-77). Ethanolamine binding to the membrane distal Cache domain triggers its compaction, and the residues corresponding to helices α3 and α4 become ordered. These conformational changes in the membrane distal Cache domain result in the anticlockwise rotation of the membrane proximal Cache domain, which is more apparent in S1 Movie. Ethanolamine is represented as spheres.

To identify ethanolamine-induced conformational changes in CqsR_p_ monomers, we aligned the α1 helices (residues 44–77) of CqsR_p_-ethanolamine and CqsR_p_-D198N (Fig 8 and S1 Movie). Within Cache monomers, this α1 helix generally undergoes little if any conformational change upon ligand-binding, and it serves as a post against which both the membrane distal and proximal Cache domains move. When ethanolamine binds to the membrane-distal Cache domain, it triggers a reorientation of residues in the binding site (Fig 8 and S1 Movie). Coincident with this movement surrounding the ligand that compacts the membrane distal Cache domain, the membrane-proximal domain rotates anticlockwise relative helix α1. The movement of the membrane-proximal domain would cause a shift in the connected transmembrane region. The absence of the transmembrane domain from the crystal structures precludes our ability to describe this change, but the relative movement of the transmembrane domains, either downward along the z-axis (like a piston) and/or a combination of xy shifts (like scissors), has been proposed to regulate the cytoplasmic histidine kinase activity of histidine kinase receptor dimers [44].

## Discussion

Ethanolamine is found in the cell membranes of all living organisms as a component of phosphotidylethanolamine [45] and fatty acids lipids such as *N*-acetylethanolamine [46], lysophosphotidylethanolamine [47], sphingomyelin [48,49], and anandamide [50]. It is also abundant in the human body, particularly in the gut, due to the constant turnover of the intestinal lumen [51–53]. The ability to sense ethanolamine for niche recognition and/or catabolize it for carbon and nitrogen is widespread among bacteria, including human commensals and pathogens [54–57].

For example, an ethanolamine utilization transcription factor upregulates ethanolamine catabolism in *Salmonella enterica* serovar Typhimurium when it enters the intestine and triggers expression of the Salmonella pathogenicity island (SPI)-1 within macrophages [58]. Ethanolamine was also demonstrated to activate virulence gene expression in Enterohemorrhagic *E. coli* O157:H7 independent of ethanolamine metabolism [59,60]. Furthermore, ethanolamine was demonstrated to regulate cytoplasmic and membrane-bound histidine sensor kinases in *Enterococcus faecalis* and *V. cholerae,* respectively [30,61,62]. In *V. cholerae*, ethanolamine binding to the periplasmic domain of the receptor CqsR controls quorum sensing and infection site selectivity in the host [30].

The most common extracellular ligand-binding domain in bacteria is the Cache domain [33]. Ligand binding to Cache domains regulates the cytoplasmic activities of their receptors, including, among others, histidine kinase, cyclic di-GMP cyclases/diesterases, chemotaxis transducers, adenylate/guanylate cyclases. The CqsR_p_-ethanolamine crystal structure reveals the mechanistic basis of ethanolamine binding to Cache domains. Central to ethanolamine binding is a highly conserved Asp, corresponding to CqsR Asp198. This residue serves as an anchor or hot spot for ligand binding, mediating hydrogen bonds to two key functional groups (hydroxyl and amine) that are optimally separated by 2 carbon atoms in the ligand (Fig 2). The interactions mediated by the platform residue are essential, as substitution with Asn resulted in the loss of *in vitro* binding and signal detection in *V. cholerae* (Figs S1 and 6, S3 Table). Additionally, the incompatibility of Asn in this position supports the requirement for an anionic Asp198 for ligand recognition through ion-pairing with a protonated amine and binding with a suitable hydrogen bond donor in the ligand.

**Fig 9:**
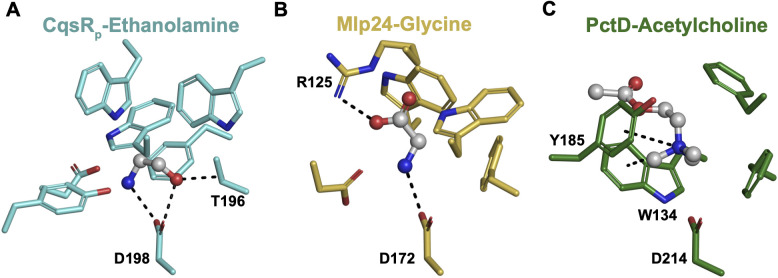
Cache domain binding modes for 2-amino alcohols, amino acids, and quaternary amines. (A) The CqsR_p_-ethanolamine binding site, highlighting the hot-spot interaction between conserved D198 and the ethanolamine amine and hydroxyl. CqsR_p_ is represented as blue sticks, ethanolamine is represented as a ball-and-stick model. (B) The Mlp24-glycine binding site, highlighting the hot-spot interaction between conserved D172 and the glycine amide as well as conserved R125 and the glycine carboxyl group. Mlp24 is represented as yellow sticks, glycine is represented as a ball-and-stick model. PDB: 6IOQ [63]. (C) The PctD-acetylcholine binding site. Quaternary amines adopt orientations driven by cation-pi bonds formed between the quaternary amine nitrogen and the side chain of an aromatic residue in the binding pocket. PctD is represented as green sticks, acetylcholine is represented as a ball-and-stick model. PDB: 7PRR [64]. Bonds noted in the discussion are depicted as black dashed lines.

Structural comparison of CqsR_p_-ethanolamine, CqsR_p_-serinol, and CqsR_p_-L-alaninol to Cache domains bound to amino acids or quaternary amines revealed important insights into the specificity of 2-amino alcohol binding to Cache domains (Fig 9). It was previously noted that amino acids bind to Cache domains in the same orientation [37], and we note that ethanolamine, serinol, and L-alaninol are bound in an essentially identical orientation to each other but quite distinct from that of the amino acid ligands. Interestingly, comparison of the CqsR_p_ ligand-bound structures (Fig 9A) and amino acid-bound Cache domain structures, for example, Mlp24-glycine (PDB 6IOQ) (Fig 9B) [63], revealed that the amide nitrogen common to all of these ligands is similarly positioned in space and bound to the platform acidic residue but that is where the orientation similarity largely ends. In the amino acid-bound Cache structures, the ligand rotates such that the carboxyl forms a hydrogen bond with a conserved positively charged residue (Fig 9B) [37]. In the structures of CqsR_p_ with ethanolamine, serinol, or L-alaninol, the ligands pivot around the amine nitrogen bound to the platform Asp198, allowing their hydroxyl to hydrogen bond with the platform Asp198 and Thr196 (Figs 2 and 5).

Interestingly, the binding of the 2-amino alcohol primary amines such as ethanolamine, serinol, and L-alaninol to the CqsR_p_ Cache domain is quite different than that of quaternary amine binding to Cache domains (Fig 9C). The structures of quaternary amines bound to Cache domains served as the foundation for the description of the Cache_1AM motif [37]. Here, it was observed that the quaternary amine ligands are oriented differently from one another within the membrane distal Cache domain ligand-binding pocket. The orientation of quaternary amines in Cache domains is driven by cation-pi bonds formed between the quaternary amine nitrogen and the side chain of an aromatic residue in the binding pocket (Fig 9C).

In sum, there is an important distinction between Cache domain binding to 2-amino alcohols, amino acids, and quaternary amines. The binding orientations of 2-amino alcohols are generally conserved, dictated primarily by their two hydrogen bonds with the platform acid residue. Similarly, the binding orientations of amino acid ligands are determined by one hydrogen bond to the platform acidic residue and another to a conserved basic residue. In contrast, quaternary amines adopt less predictable poses determined by their interaction with aromatic residues in the binding pocket.

Since Cache domains are the most common extracellular ligand-binding domain in bacteria, they are frequently central to important signaling pathways. Thus, identifying the regulatory ligand is of critical importance. Ultimately, experimental approaches are required to confirm the identity of the regulatory ligand(s), but computational approaches combining sequence analysis, structure prediction, and structural alignment to existing ligand-bound Cache structures can be used as a first pass, narrowing down the list of ligands to test experimentally.

Finally, it is important to note that while ethanolamine binds tightly to CqsR_p_ (K_d_ = 1.3 ± 0.1 μM, Fig 3A), deletion of *V. cholerae* genes essential for ethanolamine synthesis does not abolish CqsR-mediated signaling at high cell density [30]. Moreover, exogenous ethanolamine must be supplied at high concentrations to induce the high-cell-density phenotype in *V. cholerae* cultures. These findings suggest that ethanolamine primarily functions as a host-derived niche recognition signal rather than the principal quorum-sensing ligand for CqsR. Additionally, CqsR exhibits promiscuous binding, interacting strongly with the ethanolamine analogs L-alaninol and serinol, both of which similarly activate CqsR-dependent high-cell-density phenotypes in culture. We propose that ethanolamine is not the sole *V. cholerae*-derived molecule signaling through CqsR; rather, additional ligands—potentially originating from the host, other bacteria, or the environment—may also modulate CqsR activity.

## Materials and methods

### CqsR_p_ production

CqsR_p_ encoding for residues 44–260 from *V. cholerae* O1 El Tor was cloned into pTB146, resulting in pHis_6_-SUMO-CqsR_44-260_. His_6_-SUMO-CqsR_44-260_ was overexpressed in *E. coli* BL21(DE3) by growing the cells in LB medium containing 100 μg/mL ampicillin at 37°C until reaching an OD_600_ of 0.3. The temperature was then reduced to 18°C, and the culture was grown for 1 h prior to induction with 1 mM isopropyl β-D-1-thiogalactopyranoside (IPTG) for 18–20 hours at 18°C. The cells were collected by centrifugation at 4,000 rpm and resuspended in lysis Buffer A (300 mM NaCl, 50 mM HEPES (pH 8.0)) supplemented with 10% glycerol (vol/vol), 2 μg/mL DNase, and 2 μg/mL RNase. The resuspended cells were passed through a French Press at 14,000 psi and then centrifuged at 25,000 rpm for 30 min. The supernatant was incubated with His60 Ni Superflow resin (Takara) then washed with Buffer A. His_6_-SUMO-CqsR_44-260_ was eluted with Buffer A containing increasing concentrations of imidazole (50 mM – 500 mM). Protein purity was assessed using SDS-PAGE. Fractions containing His_6_-SUMO-CqsR_44-260_ were mixed with Ulp1 and dialyzed against Buffer A supplemented with 0.01% Triton. The dialysate was loaded onto a His60 Ni Superflow column to separate cleaved CqsR_44-260_ from His_6_-SUMO. Fractions containing cleaved CqsR_44-260_ were pooled and concentrated using a 10 kDa MWCO centrifugal filter at 3,500 rpm. Concentrated CqsR_44-260_ was passed through a 0.22 μM filter and loaded onto a Superdex200 (16/70) size exclusion column (Cytiva) equilibrated with Buffer B (150 mM NaCl, 20 mM HEPES (pH 8.0)). Fractions containing CqsR_44-260_ were concentrated using a 10 kDa MWCO centrifugal filter and stored at -80°C.

### Generation and purification of CqsR_p_-D198N

Substitution mutations in pHis_6_-SUMO-CqsR_44-260_ were generated using the Q5 Site-Directed Mutagenesis Kit (New England Biolabs) and the primer pairs indicated in S4 Table. DNA sequences were confirmed by whole plasmid sequencing using Oxford Nanopore Technology with custom analysis and annotation (Plasmidsaurus). CqsR_p_-D198N was purified using the protocol described for wild-type CqsR_p_.

### CqsR_p_-ethanolamine, CqsR_p_-L-alaninol, CqsR_p_-serinol, and CqsR_p_-D198N crystallization, X-ray diffraction data collection, and structure solution

Crystals of CqsR_p_ in complex with ethanolamine were obtained via the vapor diffusion method by first incubating 206.7 μM CqsR_p_ with 1.0 mM ethanolamine on ice for approximately 5 min. 1.0 μL of the CqsR_p_-ethanolamine mixture was then mixed with 1.0 μL of mother liquor containing 1.8 M lithium chloride, 0.1 M bicine (pH 9.0), and 10% (v/v) PEG 6000 (S1 Table) at 20°C to generate a 2.0 μL hanging drop. Prior to X-ray diffraction data collection, crystals were moved to a solution of mother liquor supplemented with 12% PEG 400 and 10.0 mM ethanolamine. X-ray diffraction data for CqsR_p_-ethanolamine were collected using single crystals mounted in nylon loops, and data collection occurred in a stream of dry N_2_ at 100 K. X-ray diffraction data for CqsR_p_-ethanolamine were collected at the Standford Synchrotron Radiation Lightsource (SSRL) beamline 9–2 at 0.97946 Å with a Pixel Dectris Pilatus 6M detector.

Crystals of CqsR_p_ in complex with L-alaninol were obtained via the vapor diffusion method by first incubating 206.7 μM CqsR_p_ with 10.0 mM L-alaninol on ice for approximately 5 min. 1.0 μL of the CqsR_p_-L-alaninol mixture was then mixed with 1.0 μL of mother liquor containing 1.8 M lithium chloride, 0.1 M bicine (pH 9.0), and 10% (v/v) PEG 6000 (S1 Table) at 20°C to generate a 2.0 μL hanging drop. Prior to X-ray diffraction data, crystals were moved to a solution of mother liquor supplemented with 18% PEG 400 and 10.0 mM L-alaninol. X-ray diffraction data for the CqsR_p_-L-alaninol were collected using single crystals mounted in nylon loops and data collection occurred in a stream of dry N_2_ at 100 K. X-ray diffraction data for CqsR_p_-L-alaninol was collected in-house using the Rigaku Synergy S at 1.540562 Å with a Rigaku HyPix-6000HE detector.

Crystals of CqsR_p_ in complex with serinol were obtained via the vapor diffusion method with 1.0 μL of 206.7 μM CqsR_p_ combined 1:1 with 1.0 μL mother liquor containing 1.8 M lithium chloride, 0.1 M bicine (pH 9.0), and 10% (v/v) PEG 6000 (S1 Table) at 20°C to generate a 2.0 μL hanging drop. Prior to X-ray diffraction data, crystals were moved to a solution of mother liquor supplemented with 15% PEG 400 and 10.0 mM serinol. X-ray diffraction data for CqsR_p_-serinol were collected using single crystals mounted in nylon loops, and data collection occurred in a stream of dry N_2_ at 100 K. X-ray diffraction data for CqsR_p_-serinol were collected in-house using a Rigaku Synergy S at 1.540562 Å with a Rigaku HyPix-6000HE detector.

Crystals of CqsR_p_-D198N were obtained via the vapor diffusion method with 1.0 μL of 206.7 μM CqsR_p_-D198N combined 1:1 with 1.0 μL mother liquor containing 2.0 M sodium formate and 2% (v/v) glycerol (S1 Table) at 20°C to generate a 2.0 μL hanging drop. Prior to collecting X-ray diffraction data, crystals were moved to a solution of mother liquor supplemented with 25% ethylene glycol. X-ray diffraction data for CqsR_p_-D198N were collected using single crystals mounted in nylon loops, and data collection occurred in a stream of dry N_2_ at 100 K. X-ray diffraction data for CqsR_p_-D198N were collected in-house using a Rigaku Synergy S at 1.540562 Å with a Rigaku HyPix-6000HE detector.

X-ray diffraction data for CqsR_p_-ethanolamine were processed using HKL 3000 [65]. X-ray diffraction data for CqsR_p_-L-alaninol CqsR_p_-serinol, and CqsR_p_-D198N were processed using CrysAlis PRO (Agilent (2014). Agilent Technologies Ltd, Yarnton, Oxfordshire, England).

Initial crystallographic phases were determined by molecular replacement using Phaser [66] and a CqsR_p_ search model generated using AlphaFold 3 [38] or CqsR_p_ from the CqsR_p_-ethanolamine structure. Manual model building was carried out in Coot [34] and refinement was performed in phenix.refine [67]. Initial model refinement included either automated model building or simulated annealing followed by rigid body, individual atomic coordinate, and individual B-factor refinement. Later rounds of refinement employed individual atomic coordinate, individual B-factor, and TLS refinement. TLS groups were identified using the automatic TLS selection algorithm in the Phenix selection editor. During the final rounds of refinement, the stereochemistry and ADP weights were optimized. Insufficient electron density was observed for the following residues in the flexible regions of the protein structures, and they were omitted from the model: CqsR_p_-ethanolamine 128–130; CqsR_p_-L-alaninol 128–130; CqsR_p_-serinol 111–112, 128; CqsR_p_-D198N chain A 44–45, 110–117, 128–131, 140–144, 235–237, 260, and chain B 110–115, 128–131, 141–144, 235–237, 260. Ramachandran statistics were calculated in Molprobity [68]. Molecular graphics were produced with PyMOL (PyMOL Molecular Graphics System, Version 3.0 Schrödinger, LLC).

### Microscale thermophoresis

MST ligand-binding experiments were performed using wild-type CqsR_p_ purified as described above. CqsR_p_ was labeled using the Protein Labeling Kit RED-NHS 2^nd^ Generation (NanoTemper Technologies). Absorbance readings at 205 nm, 280 nm, and 650 nm were used to determine the degree of labeling and protein concentration. 100.0 nM total CqsR_p_ (18.8 nM ligand-free CqsR_p_) in Buffer B supplemented with 0.2% Pluronic acid was mixed with ligand and incubated in the dark at room temperature for 30–45 min. Samples were then transferred to standard treated capillaries (NanoTemper Technologies) and analyzed using a NanoTemper Monolith NT.115 Blue/Red instrument at 60% LED power and medium MST power.

### CqsR_p_ LC-MS/MS

A sample of 103.3 μM purified CqsR_p_ and a 103.3 μM sample of CqsR_p_-D198N in buffer (150 mM NaCl, 20 mM HEPES, pH 8.0) were denatured at 70°C for 3 minutes and then centrifuged at 13,000 rpm for 10 minutes at 23ºC. Ethanolamine present in the supernatant from each sample was derivatized and analyzed as described previously with modifications [30]. Briefly, 50.0 μL of the resulting supernatant was mixed with 25.0 μL of 0.1 M NaHCO_3_/NaCO_2_ (pH = 9.5), 25.0 μL of acetonitrile, and 500.0 μL of freshly prepared dansyl chloride [1.8 mg/mL in acetonitrile]. The sample was heated at 60°C for 60 minutes. Then, 20.0 μL of 0.250 M NaOH was introduced and heated for an additional 10 minutes at 60°C. Afterwards, 100.0 μL of 0.425 M formic acid was incorporated and diluted 1:1 with 720.0 μL of 0.1% formic acid in water. The samples were finally filtered through a 0.22 μm PTFE syringe filter.

LC-MS/MS analysis of dansyl monoethanolamine was performed using a 6430 Triple Quadrupole LC/MS (Agilent Technologies, Santa Clara, CA, USA). A 2.0 μL aliquot of the derivatized product was injected into a Hypersil BDS C8 HPLC Column (50 x 2.1 mm x 5 μm; Thermo Fisher Scientific, Waltham, MA, USA). The mobile phases used were: A, 0.1% formic acid in HPLC grade water and B, 0.1% formic acid in acetonitrile. The column was maintained at ambient temperature with a flow rate of 0.200 mL/min. A gradient was run from 5% B to 30% B over 8 min, increased to 90% B over 1 min, and held for 2 min. The gradient returned to 5% B over 1 min and was equilibrated at 5% B for 2 min (14 min run time). Acquisition was performed in positive ion mode and the collision energy was set to 20 eV.

A MS/MS experiment was conducted to determine the fragmentation pattern and to select the appropriate product ions for monitoring. Multiple reaction monitoring (MRM) was performed using the [M + H]+ precursor ion of dansyl monoethanolamine at m/z 295 with unit mass resolution in the first quadrupole. MRM transitions of the precursor/product ions were 295/280, 295/171, and 295/157 (m/z), corresponding to the fragmentation of the precursor ion. These transitions were used to detect product ions derived from dansyl monoethanolamine. The target eluted at approximately 4.12 min for CqsR_p_ and 4.71 min for CqsR_p_-D198N. Ethanolamine concentration in each sample was inferred from ion counts using a series of pure ethanolamine standards prepared at the time of each analysis.

### Strains, media and culture conditions

All *V*. *cholerae* strains used in this study were derived from C6706str2, a streptomycin-resistant isolate of C6706 (O1 El Tor) [69]. The *lac*::P_*qrr*4_-*luxCDABE* transcriptional fusion was integrated into the genome of the strain WN5870 lacking the four quorum sensing receptors (∆*cqsS* ∆*luxPQ* ∆*cqsR* ∆*vpsS*) [30] using the plasmid pKAS32 as described [70]. To this reporter strain, CqsR or its variant was introduced on the plasmid pEVS143 under control of a P_*tac*_ promoter as previous described [30]. These CqsR variants, each with a single change at a particular amino acid residue, were constructed using site-directed mutagenesis with the plasmid pEVS143-CqsR^WT^ as the template. A complete list of strains and primers used in this study are provided in S5 and S6 Tables.

*V*. *cholerae* and *E*. *coli* cultures were grown with aeration in Luria-Bertani (LB) broth at 30°C and 37°C, respectively. Unless specified, media was supplemented with streptomycin (Sm, 100.0 μg/ml), tetracycline (Tet, 5 μg/mL), ampicillin (Amp, 100 μg/mL), kanamycin (Kan, 100 μg/mL), chloramphenicol (Cm, 5 μg/mL) and polymyxin B (Pb, 50 U/mL) when appropriate. Stock solution of ethanolamine (Sigma-Aldrich) was prepared in water and used as shown in this study.

### Bioluminescence assays

Bioluminescence assays to measure *qrr*4 transcription in different *V. cholerae* reporter strains were performed as described previously with slight modifications [11,30]. Single colonies of each strain were grown in LB broth containing appropriate antibiotics for overnight at 30°C with shaking. The overnight culture was further diluted 1000-fold in LB with antibiotics and 10.0 μM IPTG, and 200.0 μL of the diluted culture was added to each well of a black 96-well microplate with a clear bottom. Ethanolamine stocks (100X) of various concentrations was prepared in water and 2.0 μL was added to each well containing the diluted bacterial culture. Mineral oil (50.0 μL) was added to each well to prevent evaporation without a lid cover. OD_600_ and light production were measured every 30 mins for 20 hrs with shaking using a BioTek Synergy HT Plate Reader.

## Supporting information

S1 Fig Quantification of ethanolamine by LC-MS/MS analysis of the soluble fraction following CqsR_p_ and CqsR_p_-D198N denaturation. (A) Ethanolamine calibration curve for purified CqsR_p_ with triplicate measurements. The average concentration was determined to be 83.9 ± 2.6 μM for CqsR_p_ and 0.5 ± 0.2 μM for 150 mM NaCl, 20 mM HEPES, pH 8.0. (B) Ethanolamine calibration curve for CqsR_p_-D198N with triplicate measurements. The average concentration was determined to be 2.1 ± 0.2 μM in CqsR_p_-D198N and 2.1 ± 0.3 μM in 50 mM NaCl, 20 mM HEPES, pH 8.0.
(TIF)

S2 Fig CqsR_p_-D198N crystal structure. Left: The CqsR_p_-D198N monomer rendered as a yellow cartoon. Right: Expanded view of the unoccupied ligand-binding site enclosed by the box in panel A. F_o_-F_c_ electron density scaled to 3σ. No electron density corresponding to a bound ligand was observed.
(TIF)

S3 Fig CqsR_p_-D198N is unable to bind to ethanolamine, L-alaninol, and serinol. (A) Ethanolamine, (B) L-alaninol, and (C) serinol were titrated between 5.0 mM and 152.6 nM with 100.0 nM CqsR_p_-D198N.
(TIF)

S4 Fig MST quantification for CqsR_p_ binding to ethylenediamine, 2-amino-1-butanol, tris, and (S)-(+)-2-amino-1-butanol. (A) Ethylenediamine was titrated between 25.0 mM and 762.9 nM with 100.0 nM CqsR_p_. MST was performed in quadruplicate. (B) 2-amino-1-butanol was titrated between 50.0 mM and 1.5 μM with 100.0 nM CqsR_p_. MST was performed in duplicate. (C) Tris was titrated between 200.0 mM and 6.1 μM with 100.0 nM CqsR_p_. MST was performed in duplicate. (D) (S)-(+)-2-amino-1-butanol was titrated between 50.0 mM and 1.5 μM with 100.0 nM CqsR_p_. MST was performed in duplicate.
(TIF)

S5 Fig Trp138 shifts in CqsR_p_-L-alaninol and CqsR_p_-serinol to accommodate additional chemical groups. CqsR_p_-ethanolamine, CqsR_p_-L-alaninol, and CqsR_p_-serinol were structurally aligned and their carbon atoms colored cyan, orange, and green, respectively. (A) In comparison to its position in CqsR_p_-ethanolamine, Trp138 in CqsR_p_-L-alaninol shifts 0.7 Å to accommodate the additional methyl group. (B) In comparison to its position in CqsR_p_-ethanolamine, Trp138 in CqsR_p_-serinol shifts 0.6 Å to accommodate the additional methoxy group. The measurements (black dashed lines) shown in A and B are the distances from Trp138 C7 to the α-amino carbon in the respective ligand.
(TIF)

S6 Fig Structural comparison of CqsR_p_-ethanolamine to other ethanolamine-binding proteins. (A) X-ray crystal structure of CqsR_p_. (B) AlphaFold 3 model of R3_p_ (rmsd for modeled Cα carbons = 2.25 Å and 2.53 Å for pairwise comparison t*o* CqsR_p_ and R7_p_, respectively). (C) AlphaFold 3 model of R7_p_ (rmsd for modeled Cα carbons = 2.20 Å for pairwise comparison t*o* CqsR_p_). (D) The X-ray crystal structure of Csal_0678 (PDB ID 4UAB) exhibits no recognizable similarity to the tertiary structures of CqsR_p_, R3_p_, or R7_p_.
(TIF)

S7 Fig Response to exogenously added ethanolamine by different CqsR variants in V. cholerae. The data shown here are identical to those shown in Fig 6, but the data for the strain containing WT CqsR are presented using a Y-axis scale that improves the visibility of the dose-response curves. Whole-cell bioluminescence assays were performed using a ∆*cqsS* ∆*luxPQ* ∆*cqsR* ∆*vpsS* strain with different CqsR variants expressed from a plasmid. These strains also contain a P_*qrr*4_-*luxCDABE* reporter to measure the transcription of *qrr*4. Ethanolamine was added at the final concentrations as shown. Curves of the same color represent bacterial cultures grown with the same concentration of ethanolamine. For comparison, the two dotted lines on each graph show the maximum light production level of WT in the absence of ethanolamine and the minimal light production level of WT in the presence of the highest amount of ethanolamine at low cell density (OD_600_ ~ 0.2). Representative results are shown with technical duplicates. The experiments have been repeated at least three times. A biological replicate with technical triplicates is shown in S8 Fig.
(TIF)

S8 Fig Response to exogenously added ethanolamine by different CqsR variants in V. cholerae. Whole-cell bioluminescence assays were performed using a ∆cqsS ∆luxPQ ∆cqsR ∆vpsS strain with different CqsR variants expressed from a plasmid. These strains also contain a Pqrr4-luxCDABE reporter to measure the transcription of qrr4. Ethanolamine was added at the final concentrations as shown. Curves of the same color represent bacterial cultures grown with the same concentration of ethanolamine. For comparison, the two dotted lines on each graph show the maximum light production level of WT in the absence of ethanolamine and the minimal light production level of WT in the presence of the highest amount of ethanolamine at low cell density (OD_600_ ~ 0.2). Representative results are shown with technical triplicates.
(TIF)

S1 Movie CqsR_p_ morphing between the ligand-free and ethanolamine-bound conformations. The CqsR_p_ ligand-free (CqsR_p_-D198N) and ethanolamine-bound structures were aligned using their α1 helices (residues 44–77). Interpolation between the structures was performed in PyMOL (PyMOL Molecular Graphics System, Version 3.0 Schrödinger, LLC). Ethanolamine is depicted as spheres. Ethanolamine binding site residues Trp138, Tyr169, Asp171, Thr196, Asp198 are shown as sticks. D198N is depicted as aspartate throughout the morph. The residues corresponding to helices α3 and α4 are ordered only in the CqsR_p_ ethanolamine-bound structure and are not depicted in the movie.
(MPG)

S1 Table Summary of CqsR_p_ crystallization conditions at 293.15K, and cryoprotectants used at 100K^a^. (XLSX)

S2 Table LC-MS/MS Quantification of 103.3 μM purified CqsR_p_ and 150 mM NaCl 20 mM HEPES. (XLSX)

S3 Table LC-MS/MS Quantification of 103.3 μM CqsR_p_-D198N and 150 mM NaCl 20 mM HEPES. (XLSX)

S4 Table Primers used for site-directed mutagenesis. (XLSX)

S5 Table Bacterial strains used in this study. (XLSX)

S6 Table Primers used for CqsR site-directed mutagenesis. (XLSX)
