# Supplementary figures and images for: Structure-function studies of Vibrio cholerae quorum-sensing receptor CqsR signal recognition
Source: PLoS Pathog. 2025 Sep 4;21(9):e1013447. doi: 10.1371/journal.ppat.1013447 (PMC12410723; doi:10.1371/journal.ppat.1013447)

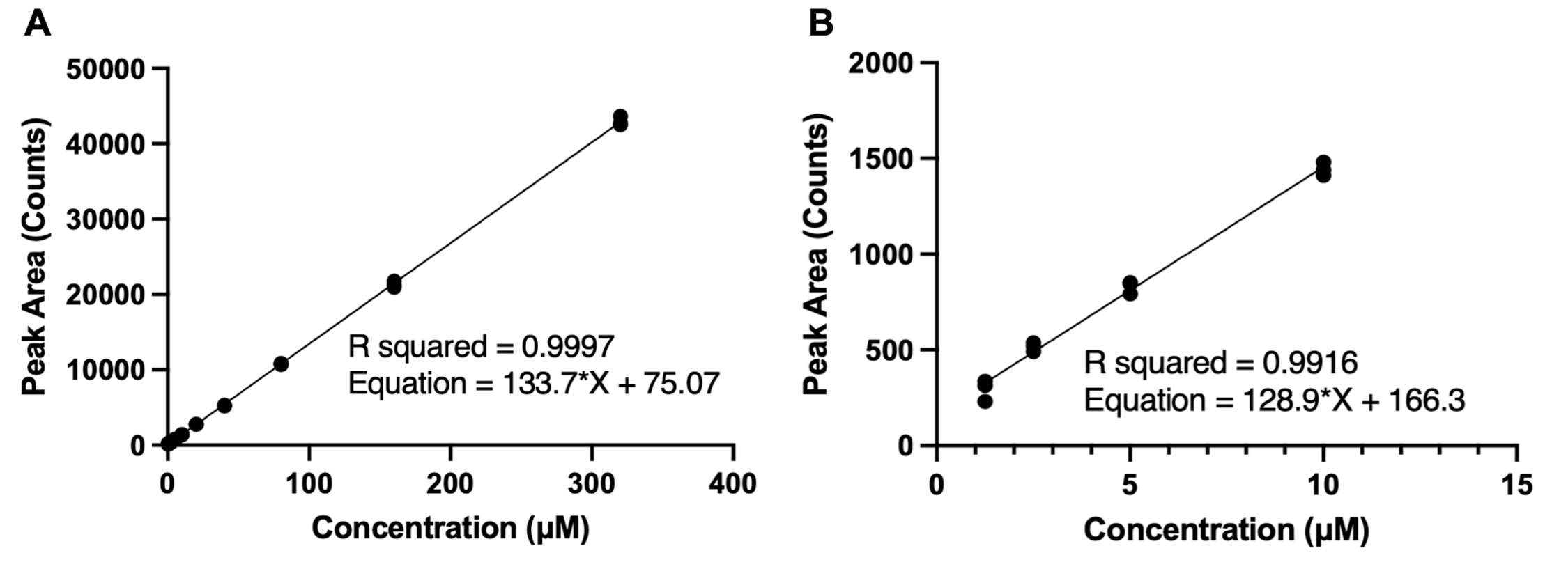

Supplement: S1 Fig — (A) Ethanolamine calibration curve for purified CqsRp with triplicate measurements. The average concentration was determined to be 83.9 ± 2.6 μM for CqsRp and 0.5 ± 0.2 μM for 150 mM NaCl, 20 mM HEPES, pH 8.0. (B) Ethanolamine calibration curve for CqsRp-D198N with triplicate measurements. The average concentration was determined to be 2.1 ± 0.2 μM in CqsRp-D198N and 2.1 ± 0.3 μM in 50 mM NaCl, 20 mM HEPES, pH 8.0. (TIF) [file ppat.1013447.s001.tif]

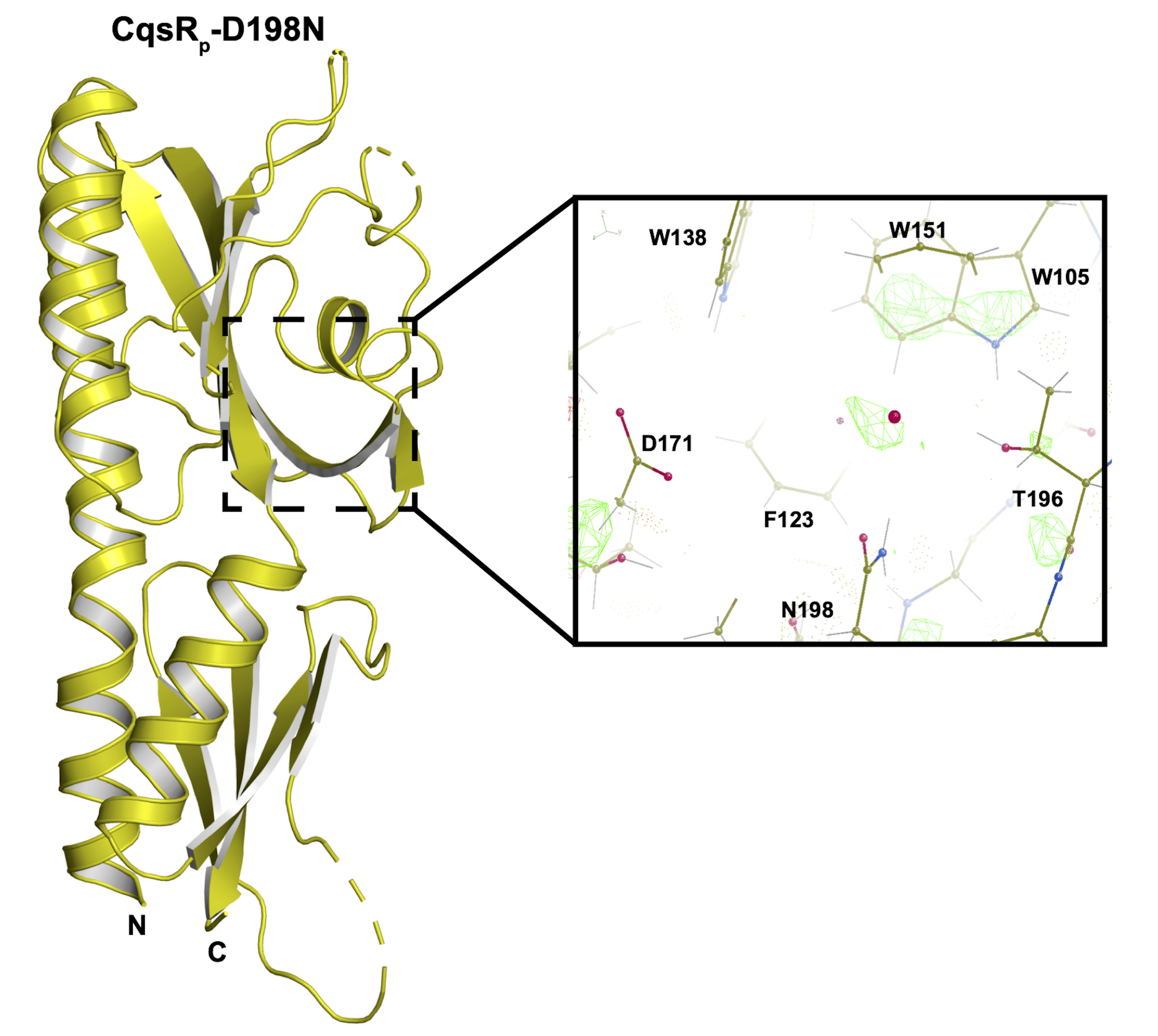

Supplement: S2 Fig — Left: The CqsRp-D198N monomer rendered as a yellow cartoon. Right: Expanded view of the unoccupied ligand-binding site enclosed by the box in panel A. Fo-Fc electron density scaled to 3σ. No electron density corresponding to a bound ligand was observed. (TIF) [file ppat.1013447.s002.tif]

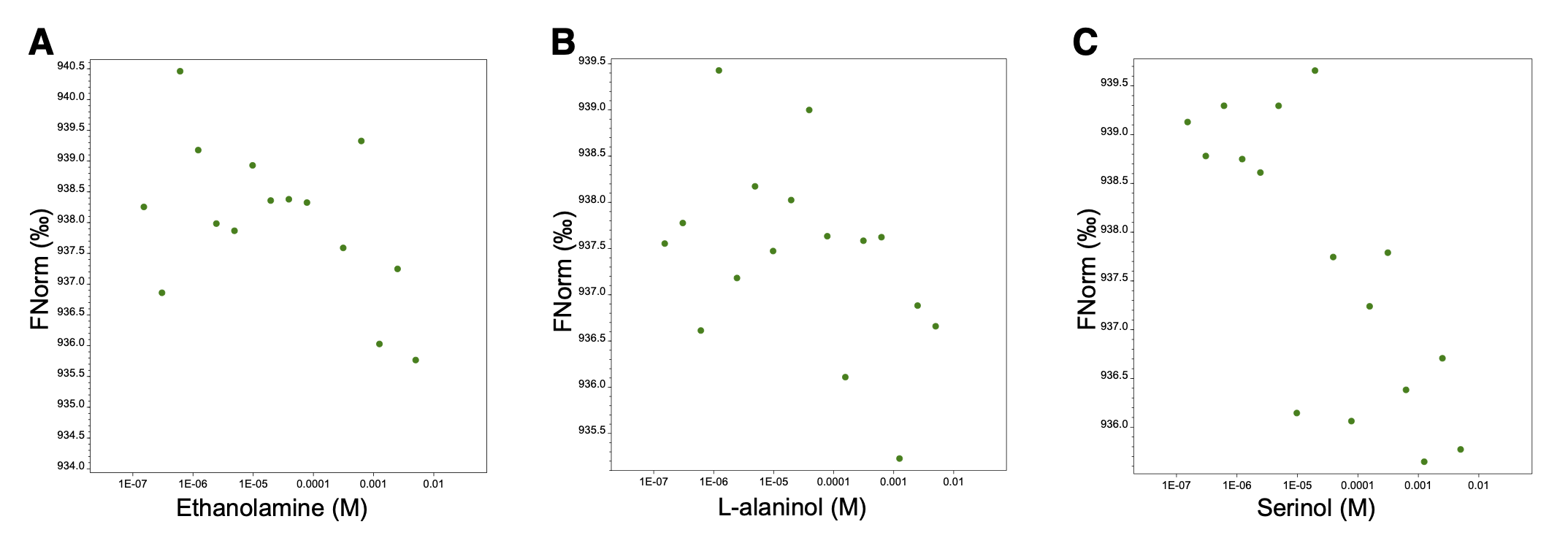

Supplement: S3 Fig — (A) Ethanolamine, (B) L-alaninol, and (C) serinol were titrated between 5.0 mM and 152.6 nM with 100.0 nM CqsRp-D198N. (TIF) [file ppat.1013447.s003.tif]

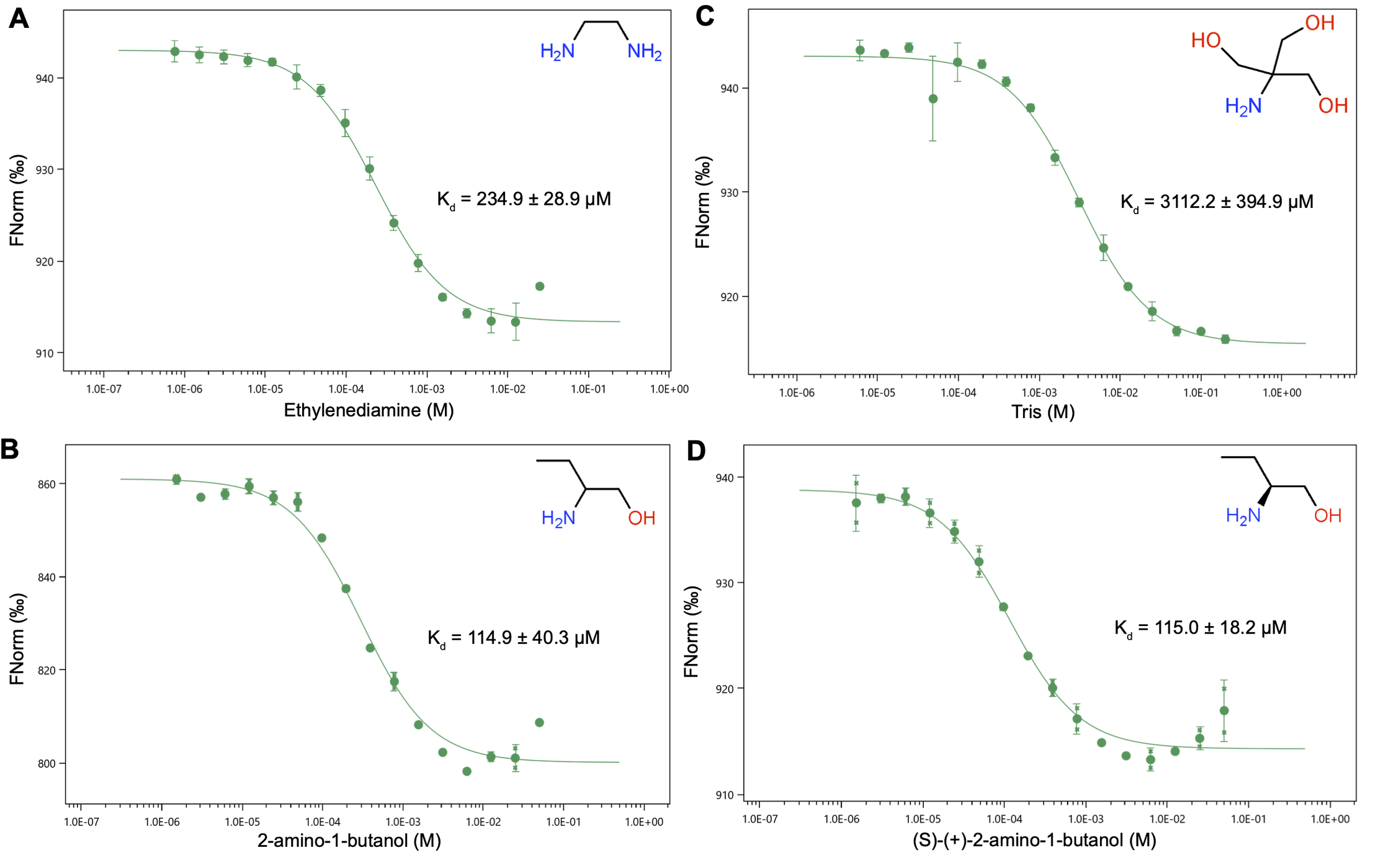

Supplement: S4 Fig — (A) Ethylenediamine was titrated between 25.0 mM and 762.9 nM with 100.0 nM CqsRp. MST was performed in quadruplicate. (B) 2-amino-1-butanol was titrated between 50.0 mM and 1.5 μM with 100.0 nM CqsRp. MST was performed in duplicate. (C) Tris was titrated between 200.0 mM and 6.1 μM with 100.0 nM CqsRp. MST was performed in duplicate. (D) (S)-(+)-2-amino-1-butanol was titrated between 50.0 mM and 1.5 μM with 100.0 nM CqsRp. MST was performed in duplicate. (TIF) [file ppat.1013447.s004.tif]

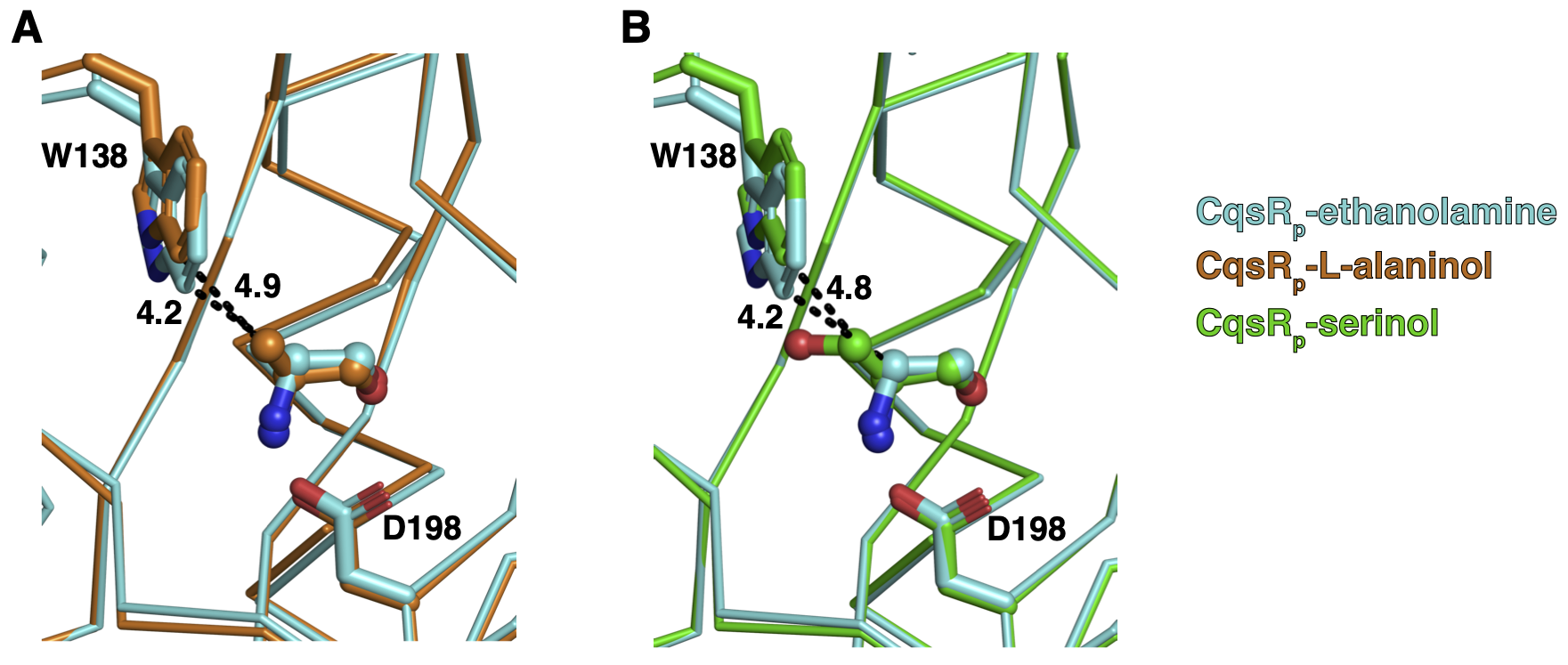

Supplement: S5 Fig — CqsRp-ethanolamine, CqsRp-L-alaninol, and CqsRp-serinol were structurally aligned and their carbon atoms colored cyan, orange, and green, respectively. (A) In comparison to its position in CqsRp-ethanolamine, Trp138 in CqsRp-L-alaninol shifts 0.7 Å to accommodate the additional methyl group. (B) In comparison to its position in CqsRp-ethanolamine, Trp138 in CqsRp-serinol shifts 0.6 Å to accommodate the additional methoxy group. The measurements (black dashed lines) shown in A and B are the distances from Trp138 C7 to the α-amino carbon in the respective ligand. (TIF) [file ppat.1013447.s005.tif]

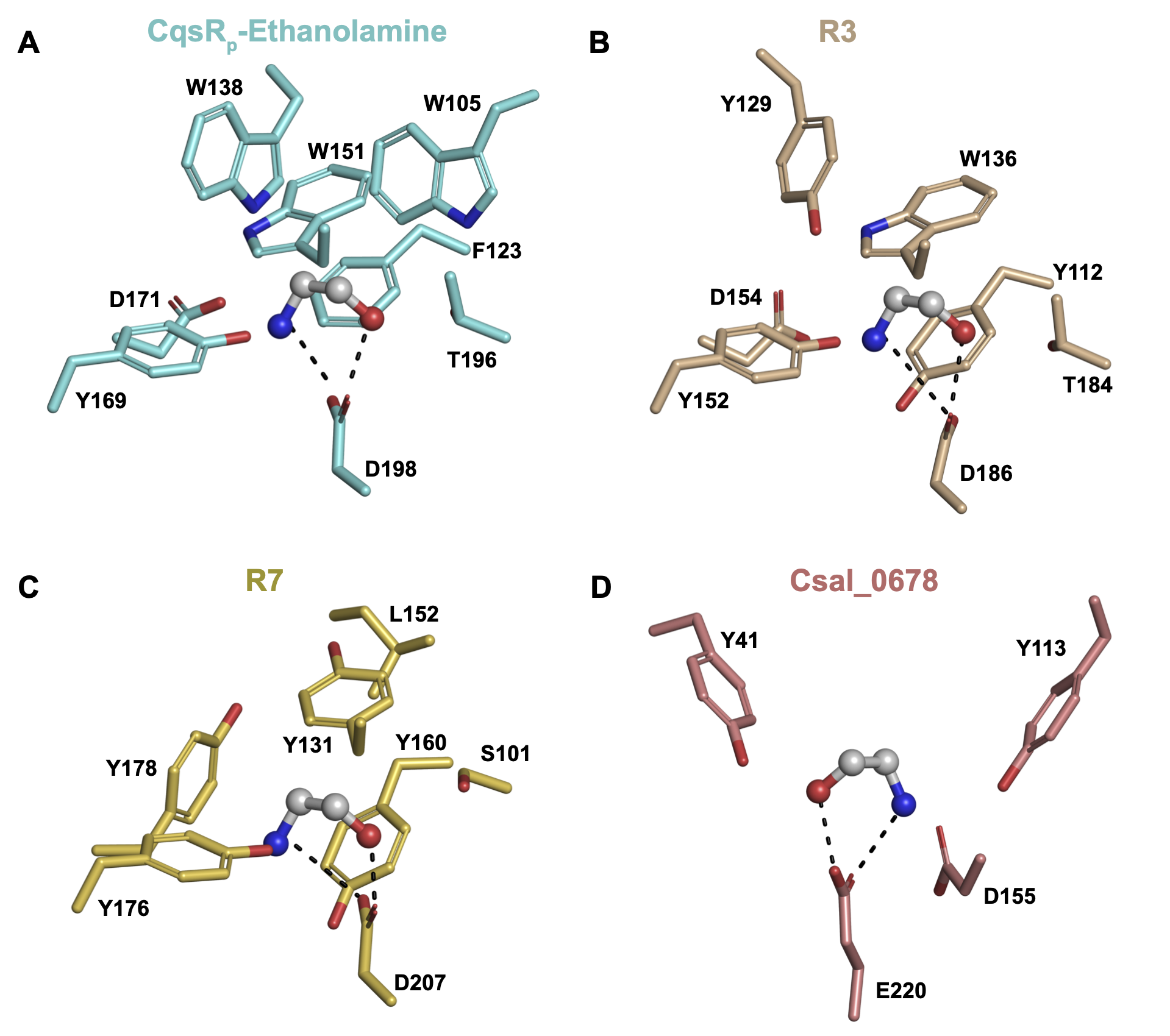

Supplement: S6 Fig — (A) X-ray crystal structure of CqsRp. (B) AlphaFold 3 model of R3p (rmsd for modeled Cα carbons = 2.25 Å and 2.53 Å for pairwise comparison to CqsRp and R7p, respectively). (C) AlphaFold 3 model of R7p (rmsd for modeled Cα carbons = 2.20 Å for pairwise comparison to CqsRp). (D) The X-ray crystal structure of Csal_0678 (PDB ID 4UAB) exhibits no recognizable similarity to the tertiary structures of CqsRp, R3p, or R7p. (TIF) [file ppat.1013447.s006.tif]

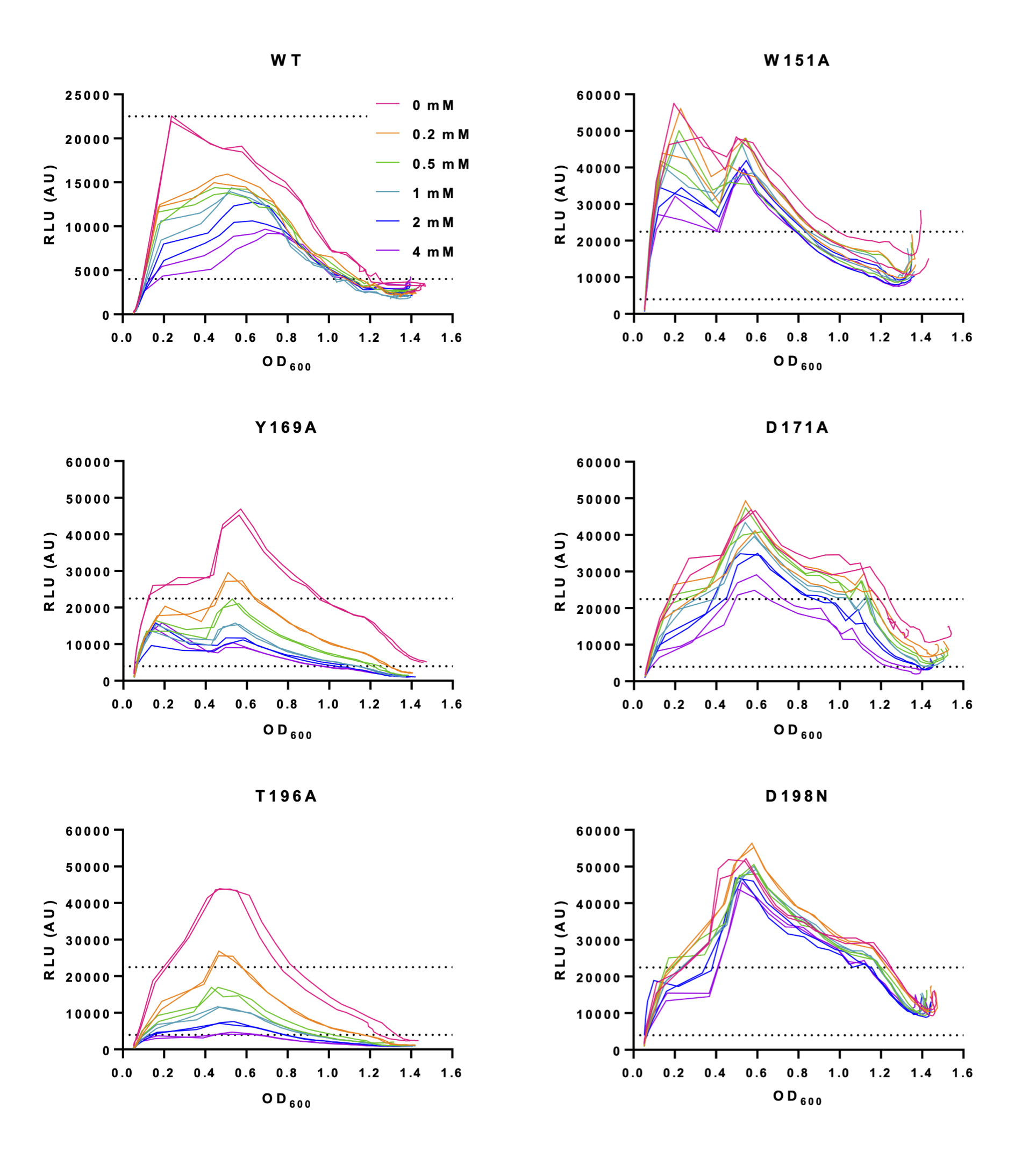

Supplement: S7 Fig — The data shown here are identical to those shown in Fig 6, but the data for the strain containing WT CqsR are presented using a Y-axis scale that improves the visibility of the dose-response curves. Whole-cell bioluminescence assays were performed using a ∆cqsS ∆luxPQ ∆cqsR ∆vpsS strain with different CqsR variants expressed from a plasmid. These strains also contain a Pqrr4-luxCDABE reporter to measure the transcription of qrr4. Ethanolamine was added at the final concentrations as shown. Curves of the same color represent bacterial cultures grown with the same concentration of ethanolamine. For comparison, the two dotted lines on each graph show the maximum light production level of WT in the absence of ethanolamine and the minimal light production level of WT in the presence of the highest amount of ethanolamine at low cell density (OD600 ~ 0.2). Representative results are shown with technical duplicates. The experiments have been repeated at least three times. A biological replicate with technical triplicates is shown in S8 Fig. (TIF) [file ppat.1013447.s007.tif]

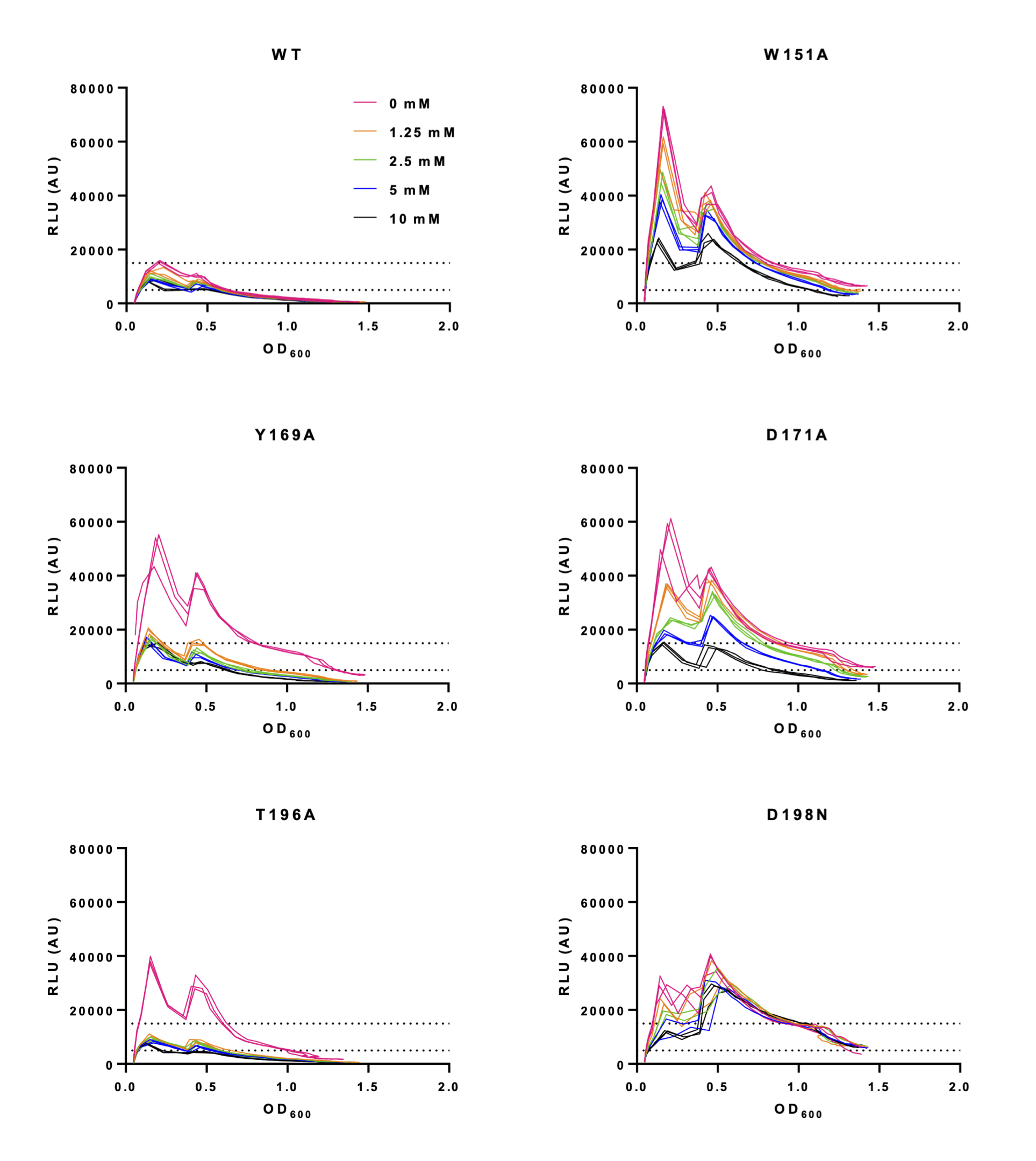

Supplement: S8 Fig — Whole-cell bioluminescence assays were performed using a ∆cqsS ∆luxPQ ∆cqsR ∆vpsS strain with different CqsR variants expressed from a plasmid. These strains also contain a Pqrr4-luxCDABE reporter to measure the transcription of qrr4. Ethanolamine was added at the final concentrations as shown. Curves of the same color represent bacterial cultures grown with the same concentration of ethanolamine. For comparison, the two dotted lines on each graph show the maximum light production level of WT in the absence of ethanolamine and the minimal light production level of WT in the presence of the highest amount of ethanolamine at low cell density (OD600 ~ 0.2). Representative results are shown with technical triplicates. (TIF) [file ppat.1013447.s008.tif]
